# CAKL: Commutative algebra k-mer learning of genomics

**Published:** 2025-08-13

**Authors:** Faisal Suwayyid, Yuta Hozumi, Hongsong Feng, Mushal Zia, JunJie Wee, Guo-Wei Wei

**Affiliations:** 1 Department of Mathematics, King Fahd University of Petroleum and Minerals, Dhahran 31261, KSA.; 2 Department of Mathematics, Michigan State University, MI 48824, USA.; 3 Department of Mathematics and Statistics, University of North Carolina at Charlotte, Charlotte, NC 28223, USA; 4 Department of Electrical and Computer Engineering, Michigan State University, MI 48824, USA.; 5 Department of Biochemistry and Molecular Biology, Michigan State University, MI 48824, USA.

## Abstract

Despite the availability of various sequence analysis models, comparative genomic analysis remains a challenge in genomics, genetics, and phylogenetics. Commutative algebra, a fundamental tool in algebraic geometry and number theory, has rarely been used in data and biological sciences. In this study, we introduce commutative algebra k-mer learning (CAKL) as the first-ever nonlinear algebraic framework for analyzing genomic sequences. CAKL bridges between commutative algebra, algebraic topology, combinatorics, and machine learning to establish a new mathematical paradigm for comparative genomic analysis. We evaluate its effectiveness on three tasks—genetic variant identification, phylogenetic tree analysis, and viral genome classification—typically requiring alignment-based, alignment-free, and machine-learning approaches, respectively. Across eleven datasets, CAKL outperforms five state-of-the-art sequence analysis methods, particularly in viral classification, and maintains stable predictive accuracy as dataset size increases, underscoring its scalability and robustness. This work ushers in a new era in commutative algebraic data analysis and learning.

## 1 Introduction

Comparative genomics examines genetic variation across species and populations to study evolution, identify functional elements, assess diversity, and reconstruct phylogeny [1, 2]. Comparative analysis of genomic sequences—such as phylogenetic inference, functional annotation, and phenotype classification [3, 4]—requires a *genome space*, a metric space whose points represent genomes and whose distances capture biologically meaningful similarities. An effective metric should reflect structural, functional, and evolutionary relationships, enabling robust comparison and downstream analyses.

Traditional approaches rely on alignment-based methods that identify substitutions, insertions, and deletions through global or local optimization. Tools such as Clustal Omega [5], MAFFT [6], and MUSCLE [7] are effective for closely related sequences [8, 9]. However, their computational cost scales poorly with sequence length and dataset size, and their accuracy deteriorates for highly divergent sequences, limiting their applicability in phylogenetics [7]. Alignment-free methods address these limitations by mapping sequences to fixed-length vectors [10], enabling scalability [11, 12] and whole-genome analysis [13, 14]. Frequency-based approaches use k-mer count vectors [15], while others employ entropy [16], Lempel–Ziv [17], or Kolmogorov complexity [18] measures. Although efficient, many neglect positional or structural information, limiting their performance in genetic variant analysis [11].

Recent advances aim to enrich such feature representations. The natural vector method (NVM) encodes statistical moments of k-mer positions [19, 20]; chaos game representation (CGR) maps sequences into fractal images [21, 22]; and Fourier power spectrum (FPS) analysis extracts dominant periodicities [23, 24]. Multi-scale integration has also been achieved via fuzzy integrals [25, 26]. Persistent challenges include sensitivity to parameter choices (e.g., k, weights, dimensions) [27, 28] and limited capacity to detect biologically significant variants, motivating novel computational approaches to genomics.

Commutative algebra, the study of commutative rings, ideals, and modules, underpins key areas of modern mathematics, including algebraic geometry, number theory, and homological algebra. Despite its foundational role in pure mathematics, it has hardly been applied in science and technology due to its abstractness and lack of metric. Recently, Suwayyid and Wei introduced multi-scale analysis to commutative algebra, enabling the potential application of abstract nonlinear algebra to data science and learning [29].

In this work, we introduce, for the first time, commutative algebra to genomics. By leveraging persistent Stanley–Reisner theory (PSRT) [29], we propose *commutative algebra k-mer learning* (CAKL) to integrate k-mers representations of sequences [30] with persistence modules arising from Stanley–Reisner constructions. CAKL is evaluated on eleven diverse datasets: one for genetic variant identification, six for phylogenetic inference, and four for viral classification derived from the National Center for Biotechnology Information (NCBI) Virus database. We systematically compare CAKL against five state-of-the-art alignment-free methods: the Natural Vector Method (NVM) [31], the Markov k-string model (MKS) [32], Jensen–Shannon (JS) divergence and Kullback–Leibler (KL) divergence, and Fourier Power Spectrum (FPS) [23] and an alignment-based method, MAFFT [6]. Section 2 presents the experimental results. Section 3 discusses performance, limitations, and generalization. Section 4 describes the proposed CAKL methodology and introduces a new purity metric for evaluating the quality of phylogenetic inference.

## 2 Results

### 2.1 An overview of CAKL

**Figure 1:**
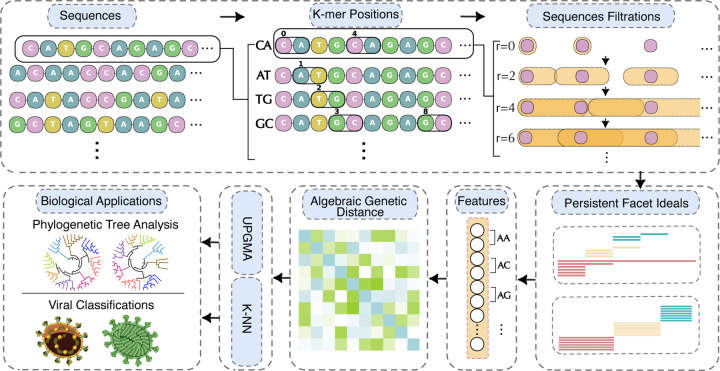
Illustration of the CAKL workflow. Given a query sequence, k-mers are first extracted. For each k-mer, the set of its occurrence positions within the sequence is treated as a sequence of integers. The persistent facet numbers associated with these sequences of integers are then computed and used to represent the corresponding k-mer. The feature vectors of all k-mers of the same size are concatenated to construct an algebraic representation. Pairwise distances between these sequences are subsequently defined and used for tasks such as genome variant identification, phylogenetic analysis, and genome classification.

To evaluate the effectiveness of the proposed CAKL method, illustrated by the workflow in Fig. 1, we consider three key applications: genetic variant identification, phylogenetic analysis, and viral classification. In the first of these, genetic variant identification is a critical application of genetics and bioinformatics that tracks genetic variations of selected gene lists associated with specific diseases, phenotypes, and populations, for which alignment methods are typically favored. Additionally, phylogenetic analysis of genetic sequences plays a fundamental role in elucidating evolutionary relationships both among and within species, where alignment-free methods have advantages [3, 4]. Finally, viral classification is a general machine learning approach for the genetic analysis and prediction of unknown viral sequences. It is challenging to design a unified approach for these diverse tasks.

### 2.2 Genetic variant identification

**Figure 2:**
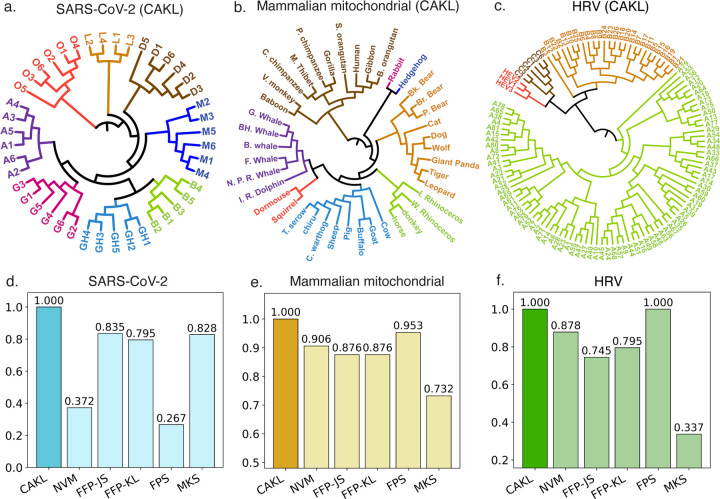
Illustration and comparison of the proposed CAKL model other methods in variant identification and phylogenetic analysis. **a.** CAKL identification of SARS-CoV-2 variants. **b–c.** CAKL phylogenetic tree analyses of mammalian mitochondrial genomes and HRV datasets. **d–f.** Comparison of prediction accuracies of different methods on the SARS-CoV-2, mammalian mitochondrial, and HRV datasets, respectively.

The emergence of SARS-CoV-2 variants during the COVID-19 pandemic posed critical challenges for monitoring viral evolution, guiding public health measures, and informing vaccine and therapeutic design [33]. Variant differences can be subtle—often just a few mutations in the spike protein receptor-binding domain [34]—across genomes of approximately 29.9 kb. For the genetic variant identification task, we analyzed 44 complete SARS-CoV-2 genomes from GISAID [30], employing CAKL with k=5 for consistency across applications. NVM was also run with k=5, while FFP-KL, FFP-JS, and Markov-based methods used k=3; FPS required no k-mer parameter. Among six alignment-free methods, only CAKL achieved perfect clustering, producing well-defined monophyletic groups (Fig. 2a, ; Supplementary Fig. S1) and accurately resolving inter-variant relationships. FFP-JS, FFP-KL, and Markov attained moderate accuracy but misclustered some Delta, Gamma, GH/490R, and Omicron samples, whereas NVM and FPS failed to produce meaningful phylogenetic structure. Performance was quantified using the label-based purity metric given by equation (24), which measures the proportion of samples with identical labels grouped under a common ancestor. Overall, CAKL achieved the highest purity score among all methods (Fig. 2d).

### 2.3 Phylogenetic analysis

Phylogenetic reconstruction is central to elucidating evolutionary relationships among taxa [3, 4]. We assessed CAKL on six benchmark datasets from [30], encompassing complete genomes and gene sequences with established taxonomic labels. Sequence lengths range from ∼2,000 nt for influenza HA genes to ∼17,000 nt for mammalian mitochondrial and Ebola virus genomes, and up to several megabases for bacterial genomes.

Phylogenetic trees were inferred using CAKL and compared with those from representative alignment-free methods. Following [30], we set k=3 for FFP-KL, FFP-JS, and Markov models, k=5 for NVM and CAKL, and omitted k for FPS. All trees were built with the UPGMA algorithm and visualized in iTOL v6 [35].

In the mammalian mitochondrial dataset, the goal was to assess how accurately each method recovers clades consistent with known host species classifications. CAKL achieved perfect concordance, producing monophyletic clades for all major mammalian orders—including delineated Primates, Cetacea, and Artiodactyla—while preserving internal coherence within Carnivora and Perissodactyla (Fig. 2b; Supplementary Fig. S2). Furthermore, CAKL attained the highest purity score among all methods (Fig. 2e).

Other methods showed varying levels of discordance: FPS partially recovered major orders but split Artiodactyla; NVM misplaced several taxa and fragmented Carnivora; FFP-JS and FFP-KL preserved many lineages but misgrouped smaller orders and split Carnivora; and MKS produced the weakest resolution, fragmenting multiple orders entirely.

In the HRV dataset, CAKL and FPS achieved perfect classification, cleanly separating HRV-A, HRV-B, and HRV-C clades, with the outgroup (HEV) distinctly isolated (Fig. 2c; Supplementary Fig. S3). NVM, FFP-JS, and FFP-KL produced moderate results, partially recovering the three clades but misplacing several HRV-A genomes within HRV-B subtrees, indicating reduced sensitivity to closely related subtypes. MKS performed poorest, failing to recover a coherent subgroup structure and producing extensive intermixing among clades, along with poor outgroup resolution.

In the HEV dataset, CAKL, FFP-JS, FFP-KL, and NVM all achieved perfect genotypic clustering (Supplementary Fig. S4). Markov misclassified one Group 3 genome, while FPS performed worst, failing to separate Groups 3 and 4, though correctly clustering Group 1.

In the influenza HA dataset, CAKL, FFP-JS, and FFP-KL all achieved perfect subtype classification (Supplementary Fig. S5), though FFP-JS and FFP-KL grouped H3N2 and H2N2 under a shared node, unlike CAKL. NVM failed to cluster H1N1 cohesively and misclassified one H2N2 sequence, while Markov misclassified an H1N1. FPS performed worst, correctly clustering only the H7N9 subtype.

In the ebolavirus dataset, all methods correctly separated the five known species (Supplementary Fig. S6). NVM, FFP-JS, FFP-KL, and Markov distinguished epidemic lineages except for the 1996 outbreak, with NVM producing the longest inter-clade branches. FFP-JS and FFP-KL showed shorter branches, indicating weaker sensitivity, while Markov grouped EBOV and RESTV under a common ancestor. FPS failed to resolve the epidemic-level structure within EBOV.

To evaluate scalability, we applied CAKL to 30 complete bacterial genomes ranging from 0.9 to 6.5 Mb. Despite the substantial increase in sequence size and complexity, all methods except NVM and FPS recovered correct family-level groupings (Supplementary Fig. S7). Overall, CAKL consistently delivered accurate and biologically coherent phylogenies, outperforming existing alignment-free methods across diverse genomic datasets.

### 2.4 Viral classification

In this task, we conduct viral classification experiments on the four NCBI datasets described in Supplementary Section S1, following the experimental design outlined by Hozumi et al. [30]. In particular, we adopt the same comparative benchmarking strategies to ensure consistency. The labels assigned to viral sequences in the NCBI Virus Database are regularly revised, as the International Committee on Taxonomy of Viruses (ICTV) continuously updates viral classifications based on new scientific findings. This ongoing process reflects the complexity of the classification problem and emphasizes the importance of using methods that remain reliable despite changes in biological taxonomy.

Two classification tasks are considered. The first employs a 1-nearest neighbor (1-NN) classifier on feature representations derived from the persistent facet ideals featurization, following the methodology introduced by Sun et al. [31]. A test sample is deemed correctly classified if its nearest neighbor, under the algebraic distance metric, shares the same viral family label. This protocol models realistic scenarios in which newly sequenced viral genomes are annotated based on proximity to previously characterized reference genomes.

The second task involves a 5-nearest neighbors (5-NN) classifier, using the same experimental protocol described in [30]. Performance is assessed via 5-fold cross-validation repeated over 30 random seeds to ensure statistical robustness. To control for class imbalance and maintain classification reliability, the evaluation is restricted to viral families with at least 15 representative sequences. In both tasks, we empirically observed that increasing the value of k in the k-mer algebraic representations generally leads to a decline in model performance. This behavior is expected, as larger values of k result in more distinct representations for each genome, irrespective of their biological classification, thereby reducing the amount of shared structural information that can be effectively used for grouping. The model exhibits consistently strong performance for k=3, 4, 5, with k=4 selected for use in both tasks.

For each dataset, stratified 5-fold cross-validation was conducted using 30 independent random seeds to obtain performance metrics. Classification performance was assessed using accuracy (ACC), balanced accuracy (BA), macro-F1 score (F1), recall, and precision. All metrics were computed using the macro-averaging scheme to ensure equal weight across viral families, regardless of class imbalance.

All methods exhibit a consistent decrease in accuracy from 2020 to 2024 across both classification tasks. As summarized in Table 1, our model exhibits a strong predictive performance under the 1-nearest neighbor (1-NN) classification protocol. On the NCBI 2020 dataset, consisting of 6,993 samples, the model achieved an accuracy of 0.932. For the larger NCBI 2022 dataset comprising 11,428 samples, an accuracy of 0.920 was obtained. For the NCBI 2024 dataset, we evaluated performance under two settings: for the filtered set of 12,154 samples, an accuracy of 0.891 was obtained, while for the complete set containing 13,645 samples, the model achieved an accuracy of 0.892.

**Figure 3:**
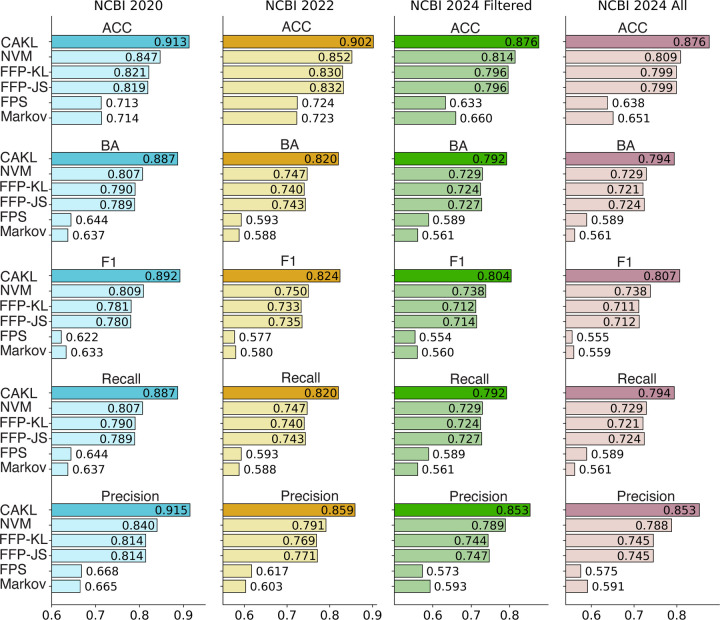
Comparison of 5-NN classification scores of the six methods, where the scores of NVM, FFP-JS, FFP-KL, FPS and Markov are obtained from [30].

Furthermore, using 5-nearest neighbors classification with 5-fold cross-validation, as summarized in Fig. 3 and Supplementary Table S2, our proposed model demonstrates higher predictive performance compared to existing state-of-the-art approaches across all datasets. Specifically, on the NCBI 2020 dataset, our model achieved an accuracy of 0.913. On the larger NCBI 2022 dataset, the model attained an accuracy of 0.902. On the filtered NCBI 2024 dataset, the model achieved an accuracy of 0.876, while on the full NCBI 2024 dataset containing all samples, an accuracy of 0.876 was recorded. These results underscore the robustness and effectiveness of our method in modeling complex viral genome spaces and establishing reliable predictive frameworks for viral classification tasks.

## 3 Discussion

### Overall performance.

Across all benchmark evaluations, CAKL demonstrates consistently superior performance over each of the five baseline alignment-free methods. On six phylogenetic tree–construction datasets, it achieves the highest tree accuracy, with the purity index (24) exceeding that of every competitor by at least 4 percentage points (absolute difference; Fig. 2). On the four extensive NCBI collections curated in [27, 30, 31], CAKL attains the top macro–averaged scores for accuracy (ACC), balanced accuracy (BA), $F_{1}$, recall, and precision, surpassing the runner-up by 4–7 percentage points across all datasets (Fig. 3).

This improvement stems from CAKL’s explicit encoding of the spatial distribution of k-mers through locality-sensitive features, in contrast to most alignment–free methods, which rely primarily on k-mer frequency distributions with limited positional or spatial information. This positional awareness enables CAKL to resolve subtle yet biologically consequential sequence variants, thereby enhancing performance in large-scale viral classification, as well as in phylogenetic inference and genetic variant identification.

### Comparable to alignment methods for variant inference

Alignment-based tools such as MAFFT excel on variant sequences of the same specie but they do not work for multi-species that cannot be aligned. To compare CAKL and MAFFT under conditions favorable to alignment methods, we analyzed three benchmark datasets—SARS-CoV-2, mammalian mitochondrial genomes, and human rhinovirus (HRV). The trees produced by CAKL and MAFFT show great similarities: both recover the principal SARS-CoV-2 clades (A, B, GH, G, O, D, L, M), correctly group *Primates*, *Carnivora*, and *Cetacea* in the mammalian set, and delineate HRV-A, HRV-B, HRV-C with the HEV outgroup (Fig. 2, Supplementary. Fig. S8). Hence CAKL attains sufficient alignment-level accuracy where alignment is presumed strongest while retaining robustness and efficiency for heterogeneous or multi-specie data, establishing it as a competitive alignment-free engine for variant inference.

### Robustness.

CAKL maintains high accuracy as the dataset size and quality vary. In a 5–NN setting, its accuracy declines only modestly, from 91.3% on the NCBI 2020 dataset consisting of 6993 sequences to 87.6% on the 13 645-sequence NCBI 2024 All dataset, exhibiting similar stability when error-containing reads are included. A 1–NN evaluation shows the same pattern, with performance decreasing from 93.2% to 89.2% across the same data progression. Empirically, $k\in \left\{3,4,5\right\}$ suffices for strong results, with k=4 giving the best single-model accuracy; combining these values in an ensemble further boosts performance with negligible additional cost.

### Interpretability of CAKL.

**Figure 4:**
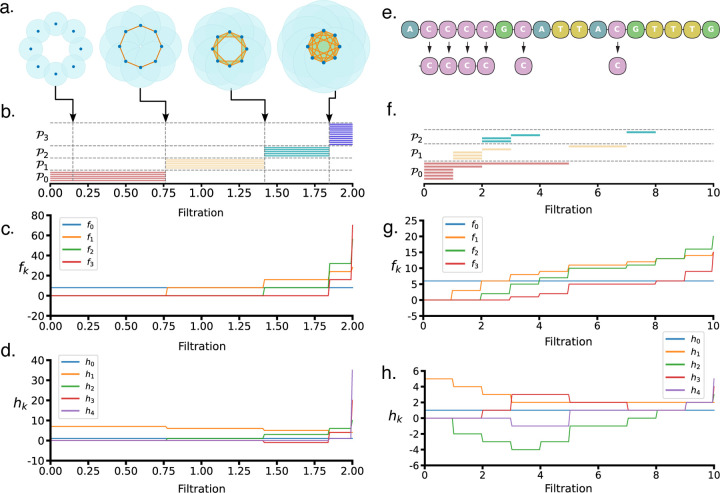
Illustrative Example of Persistent Homology and Persistent Stanley–Reisner Invariants. **a.** A filtration of a simplicial complex arising from an octagon. **b.** The persistent facet ideal barcodes derived from the same filtration, encoding combinatorial face-level activity rather than homology. **c-d.** The persistent f-vector curves, and the persistent h-vector curves derived from the same filtration of the simplicial complex, respectively. **e.** The N1-U.S-P primer sequence, with the positions of the nucleotide C marked. **f.** The persistent facet ideal barcodes of the positions, reflecting the activity of the persistent facet ideals under the induced filtration. **g-h.** The persistent f-vector curves, and the persistent h-vector curves derived from the same filtration of the sequence, respectively.

Rational learning requires explainable features and an interpretable neural network design. Fig. 4 illustrates the interpretability of CAKL through two representative examples, demonstrating how the Persistent Stanley–Reisner Theory (PSRT) encodes the filtration structure via the algebraic decomposition of facet ideals. Panels (a–d) depict a synthetic example of eight points uniformly placed on a circle of radius 1. As the filtration parameter increases, simplicial complexes are constructed by adding a k-simplex whenever all its vertices lie within the closed ball centered at at least one of its vertices. Panel (b) shows the barcodes of persistent facet ideals: $P_{0}$ encodes vertex lifespans (isolation), $P_{1}$ tracks edge persistence until subsumed into higher simplices, and $P_{2}$, $P_{3}$ capture 2- and 3-simplices respectively. The barcodes reveal uniform connectivity and regular geometric structure in this example.

Panels (e–f) present a biologically motivated example, examining the distribution of the 1-mer C in the N1-U.S-P primer. Each occurrence of C is treated as an integer in 1D space. Equal-length bars in $P_{0}$ reflect regularly spaced cytosines, while longer bars indicate isolated ones. Notably, one isolated C connects to its nearest neighbor at filtration radius 5, and its corresponding edge vanishes at radius 7, suggesting nearby higher-order interactions. The brief lifespans in $P_{2}$ indicate that 2-simplices (triangles) are formed quickly and filled soon thereafter, implying tight local clustering among cytosines.

Panels (c) and (g) show the persistent f-vectors, $f_{k}\left(r\right)$, quantifying the number of active k-simplices at filtration value r, while Panels (d) and (h) show the corresponding h-vectors, $h_{k}\left(r\right)$, measuring the incremental additions of independent k-facets. In both examples, increases in $f_{1}$ signal edge formation, and any growth in $f_{k}$ for k≥2 is necessarily preceded by growth in $f_{1}$, making $f_{1}$ a necessary precursor for higher-order structure. The h-vectors further reveal when such structures are nontrivial versus when they are absorbed into larger simplices.

Altogether, CAKL’s interpretability stems from its ability to encode k-mer spatial configurations as algebraic signatures derived from the evolution of facet ideals across the filtration. The persistent f- and h-vectors provide structured summaries of how generators of these ideals emerge and interact at varying scales. In particular, they capture the combinatorial and geometric regularity of k-mer distributions, offering insights into clustering, isolation, and interaction patterns among sequence motifs in both synthetic and biological settings.

### Generalizability.

Because CAKL encodes a sequence purely as a word over a finite alphabet, it extends naturally beyond the DNA datasets analysed here. In practice, we transcribed RNA sequences to their DNA equivalents, but the same framework accommodates amino-acid strings for protein sequence modeling. More broadly, any categorical sequence data built from a limited alphabet of letters can be modeled by the proposed commutative-algebraic constructions. The proposed CAKL can furnish a versatile mathematical foundation for sequence analysis and, by extension, for data science tasks that involve symbolic or ordered data.

## 4 Methods

In this section, we provide an overview of persistent Stanley–Reisner theory. Then, we provide the construction of the k-mer algebraic representations of sequences. We also propose a new purity metric to assessing the performance phylogenetic analysis tools.

### 4.1 Persistent Stanely–Reisner theory

Persistent Stanley–Reisner theory is a novel framework for algebraic data analysis, leveraging tools from commutative algebra. Unlike traditional topological data analysis, which emphasizes geometric and topological features, such as loops and voids, through persistent homology [36], persistent Stanley–Reisner theory focuses on the algebraic and combinatorial structure of simplicial complexes, using invariants derived from commutative algebra[29]. A filtration process is then applied to these complexes to track the evolution and persistence of such features across multiple spatial or geometric scales. This approach introduces algebraic invariants such as persistent h-vectors, f-vectors, graded Betti numbers, and facet ideals, thus providing a new algebraic perspective within the broader framework of algebraic data analysis.

#### 4.1.1 Persistent Stanley–Reisner structures over a filtration

Let k be a field, and let Δ be a simplicial complex on the finite vertex set $V=\left\{x_{1},\ldots ,x_{n}\right\}$. Suppose f:Δ→ℝ is a monotone function, i.e., $f\left(\tau \right)\leq f\left(\sigma \right)$ whenever τ⊆σ, which induces an increasing filtration

$$\tilde{f}:={\left(\Delta^{t}\right)}_{t\in \mathbb{R}},\;\;\;\text{where}\;\;\;\Delta^{t}:=\left\{\sigma \in \Delta \left|f\left(\sigma \right)\leq t\right.\right\}.$$

Let $S=k\left[x_{1},\ldots ,x_{n}\right]$ be the standard graded polynomial ring over k, and for each t∈ℝ, define the Stanley–Reisner ideal of $\Delta^{t}$ as

$$I^{t}:=\langle x_{i_{1}}\cdots x_{i_{r}}\left|\left\{x_{i_{1}},\ldots ,x_{i_{r}}\right\}\notin \Delta^{t}\right.\rangle \subseteq S,$$

with corresponding Stanley–Reisner ring

$$k\left[\Delta^{t}\right]:=S/I^{t}.$$

Since the filtration is increasing, the subcomplexes satisfy $\Delta^{s}\subseteq \Delta^{t}$ for s≤t, which implies a descending chain of monomial ideals:

$$I^{s}⊇I^{t}\;\;\;\text{for}\;\text{all}\;\;s\leq t.$$

Each ideal $I^{t}$ admits a canonical primary decomposition indexed by the facets of $\Delta^{t}$:

(1) $$I^{t}=\underset{\sigma \in F\left(\Delta^{t}\right)}{\cap }P_{\sigma },\;\;\;\;\;\text{where}\;\;\;P_{\sigma }:=\left(x_{i}\left|x_{i}\notin \sigma \right.\right).$$

We refer to the collection $P^{t}:=\left\{P_{\sigma }\left|\sigma \in F\left(\Delta^{t}\right)\right.\right\}$ as the facet ideals of $\Delta^{t}$.

To capture the dimension-wise structure, we stratify by face dimension: for each i≥0, where we define

(2) $$P_{i}^{t}:=\left\{P_{\sigma }\in P^{t}\left|\dim\left(\sigma \right)=i\right.\right\},$$

so that

$$P^{t}=\overset{\dim\left(\Delta^{t}\right)}{\underset{i=0}{∐}}P_{i}^{t}.$$

We define persistence algebraically as follows: a facet ideal $P_{\sigma }\in P_{i}^{t}$ is said to persist to level $t^{\prime }>t$ if $P_{\sigma }\in P_{i}^{t^{\prime }}$. The set of such persistent i-dimensional primes is

(3) $$P_{i}^{t,t^{\prime }}:=P_{i}^{t}\cap P_{i}^{t^{\prime }}.$$

The corresponding facet persistent number is given by

(4) $$F_{i}^{t,t^{\prime }}:=\left|P_{i}^{t,t^{\prime }}\right|,$$

which records the number of i-dimensional prime components common to $\Delta^{t}$ and $\Delta^{t^{\prime }}$.

The collection ${\left\{F_{i}^{t,t^{\prime }}\right\}}_{i,t,t^{\prime }}$ serves as a combinatorial invariant encoding the persistence of prime facets in the Stanley–Reisner filtration, providing an algebraic analogue of topological barcodes in persistent homology.

#### 4.1.2 Persistent graded Betti numbers of Stanley–Reisner rings

Let k be a field and $S=k\left[x_{1},\ldots ,x_{n}\right]$ the standard graded polynomial ring. For each filtration level t∈ℝ, the Stanley–Reisner ring $k\left[\Delta^{t}\right]:=S/I^{t}$ inherits a natural ℤ-graded S-module structure and admits a minimal graded free resolution:

(5) $$\cdots \to \underset{j}{⊕}S{\left(-j\right)}^{\beta_{i,j}\left(k\left[\Delta^{t}\right]\right)}\to \cdots \to k\left[\Delta^{t}\right]\to 0,$$

where $\beta_{i,j}\left(k\left[\Delta^{t}\right]\right):=\dim_{k}{\text{Tor}}_{i}^{S}{\left(k\left[\Delta^{t}\right],k\right)}_{j}$ are the graded Betti numbers.

Hochster’s formula relates these graded Betti numbers to the topological Betti numbers of the induced subcomplexes:

(6) $$\beta_{i,j+i}\left(k\left[\Delta^{t}\right]\right)=\sum_{\begin{array}{l} \;W\subseteq V\\ \left|W\right|=j+i \end{array}}\dim_{k}{\tilde{H}}_{j-1}\left(\Delta_{W}^{t};k\right),$$

where ${\tilde{H}}_{j-1}\left(\Delta_{W}^{t};k\right)$ denotes the $\left(j-1\right)$-st reduced simplicial homology group over k, and $\Delta_{W}^{t}:=\left\{\sigma \in \Delta^{t}\left|\sigma \subseteq W\right.\right\}$ is the subcomplex induced on the vertex set W⊆V.

In particular, Hochster’s formula can be reformulated in terms of the (non-reduced) Betti numbers of induced subcomplexes. For each integer i≥0, the following identities hold:

(7) $$\beta_{i,i+1}\left(k\left[\Delta^{t}\right]\right)=\sum_{\begin{array}{l} \;W\subseteq V\\ \left|W\right|=i+1 \end{array}}\left(\beta_{0}\left(\Delta_{W}^{t}\right)-1\right),$$

(8) $$\beta_{i,i+j}\left(k\left[\Delta^{t}\right]\right)=\sum_{\begin{array}{l} \;W\subseteq V\\ \left|W\right|=i+j \end{array}}\beta_{j-1}\left(\Delta_{W}^{t}\right),\;\;\;\;\;\text{for}\;\text{all}\;\;j\geq 2,$$

where $\Delta_{W}^{t}$ denotes the subcomplex of $\Delta^{t}$ induced on the vertex subset W⊆V, and $\beta_{r}\left(\Delta_{W}^{t}\right)$ denotes the r-th Betti number of $\Delta_{W}^{t}$ with coefficients in k.

To refine this in a persistent setting, for $t\leq t^{\prime }$, we define the persistent graded Betti number

(9) $$\beta_{i,i+j}^{t,t^{\prime }}\left(k\left[\Delta \right]\right):=\sum_{\begin{array}{l} \;W\subseteq V\\ \left|W\right|=i+j \end{array}}\dim_{k}\left(\iota_{j-1}^{t,t^{\prime }}:{\tilde{H}}_{j-1}\left(\Delta_{W}^{t}\right)\to {\tilde{H}}_{j-1}\left(\Delta_{W}^{t^{\prime }}\right)\right),$$

where $\iota_{j-1}^{t,t^{\prime }}$ is the homomorphism on reduced homology induced by inclusion. This provides a multigraded algebraic refinement of classical persistent Betti numbers, encoding both topological persistence and the combinatorial properties of the evolving homology classes.

In the special case where $\left|W\right|=\left|V\right|$, the persistent graded Betti number reduces to

$$\beta_{i,\left|V\right|}^{t,t^{\prime }}=\beta_{\left|V\right|-i-1}^{t,t^{\prime }},$$

recovering the classical persistent Betti number of homological degree $\left|V\right|-i-1$. More generally, the family ${\left\{\beta_{i,i+j}^{t,t^{\prime }}\right\}}_{i,j}$ encodes a richer multiscale invariant that interpolates between algebraic and topological persistence.

#### 4.1.3 Persistent f- and h-vectors

Let ${\left(\Delta^{t}\right)}_{t\in \mathbb{R}}$ be a filtration of a finite $\left(d-1\right)$-dimensional simplicial complex Δ, induced by some face function f:Δ→ℝ. For each fixed level t, the complex $\Delta^{t}$ consists of those faces σ∈Δ with $f\left(\sigma \right)\leq t$. At each level t, one may associate the classical combinatorial invariants of face counts and their derived quantities.

The f-vector of $\Delta^{t}$ is defined as

$$f\left(\Delta^{t}\right)=\left(f_{-1}^{t},f_{0}^{t},f_{1}^{t},\ldots ,f_{d-1}^{t}\right),$$

where $f_{i}^{t}$ denotes the number of i-dimensional faces in $\Delta^{t}$, $f_{-1}^{t}=1$ by convention, and $d=d\left(t\right)=\dim\left(\Delta^{t}\right)+1$. The associated h-vector is defined as $h\left(\Delta^{t}\right)=\left(h_{0}^{t},\ldots ,h_{d}^{t}\right)$, where

(10) $$h_{m}^{t}=\sum_{j=0}^{m}\left(\begin{array}{c} \begin{array}{c} d\left(t\right)-j\\ m-j \end{array} \end{array}\right){\left(-1\right)}^{m-j}f_{j-1}^{t},\;\;\;\;\;\text{for}\;\;m=0,\ldots ,d\left(t\right),$$

and $h_{m}^{t}=0$ for all $m>d\left(t\right)$.

This transformation is invertible, with the inverse relation given by

(11) $$f_{m-1}^{t}=\sum_{i=0}^{m}\left(\begin{array}{c} \begin{array}{c} d\left(t\right)-i\\ m-i \end{array} \end{array}\right)h_{i}^{t},\;\;\;\;\;\text{for}\;\;m=0,\ldots ,d\left(t\right),$$

where $d\left(t\right)=\dim\left(\Delta^{t}\right)+1$.

To extend these invariants to the persistent setting, one replaces the classical Betti numbers with the persistent graded Betti numbers $\beta_{i,j}^{t,t^{\prime }}$ defined over filtration levels $t\leq t^{\prime }$ [29]. This enables a multiscale, combinatorial interpretation of how face structures persist across different filtration levels.

Let ${\left(\Delta^{t}\right)}_{t\in \mathbb{R}}$ be a filtration of a simplicial complex Δ. The persistent *h*-vector between levels $t\leq t^{\prime }$ is defined as

(12) $$h_{m}^{t,t^{\prime }}:=\sum_{j=0}^{m}\left(\begin{array}{c} n-d\left(t^{\prime }\right)+m-j-1\\ m-j \end{array}\right)\left(\sum_{i=0}^{j}{\left(-1\right)}^{i}\beta_{i,j}^{t,t^{\prime }}\right),\;\;\;\;\;\text{for}\;\;m=0,\ldots ,d\left(t^{\prime }\right),$$

where $\beta_{i,j}^{t,t^{\prime }}$ denotes the persistent graded Betti numbers of $k\left[\Delta \right]$ over $\left[t,t^{\prime }\right]$, and let $d\left(t^{\prime }\right)=\dim\left(\Delta^{t}\right)+1$.

The corresponding persistent f-vector is then defined via the inverse transformation:

(13) $$f_{m-1}^{t,t^{\prime }}:=\sum_{i=0}^{m}\left(\begin{array}{c} d\left(t^{\prime }\right)-i\\ m-i \end{array}\right)h_{i}^{t,t^{\prime }},\;\;\;\;\;\text{for}\;\;m=0,\ldots ,d\left(t^{\prime }\right).$$

These persistent vectors capture how the combinatorial structure of Δ evolves through the filtration, blending face enumeration with homological persistence. In contrast to the classical static f- and h-vectors, their persistent counterparts reflect the dynamic appearance and disappearance of faces and their relations across multiple scales, providing richer algebraic-combinatorial invariants for analysis.

We now consider the following simplifications, which will play a central role in the k-mer algebraic representation framework. These observations refine the relationship between persistent h-vectors and persistent graded Betti numbers, serving to streamline computations in applications involving Vietoris–Rips complexes derived from sequence data.

Let $\beta_{i,j}^{t,t^{\prime }}$ denote the persistent graded Betti numbers of the Stanley–Reisner ring $k\left[\Delta \right]$ over the filtration interval $\left[t,t^{\prime }\right]$, as defined in equation (9). To streamline notation, we set

(14) $$B_{j}:=\sum_{i=0}^{j}{\left(-1\right)}^{i}\beta_{i,j}^{t,t^{\prime }}.$$

Alongside this, one introduces the coefficients

(15) $$\alpha_{j}^{\left(m\right)}:=\left(\begin{array}{c} n-d\left(t^{\prime }\right)+m-j-1\\ m-j \end{array}\right),$$

which appear in the linear transformation relating the h-vector of a simplicial complex to its graded Betti numbers.

It follows that the persistent h-vector component $h_{m}^{t,t^{\prime }}$ satisfies the identity

(16) $$h_{m}^{t,t^{\prime }}=\sum_{j=0}^{m}\alpha_{j}^{\left(m\right)}B_{j},\;\;\;\;\;\text{for}\;\;\text{each}\;\;m\in \mathbb{N}.$$

Additional structural identities among the persistent Betti numbers further simplify this formula. In particular, it is known that

$$\beta_{0,0}^{t,t^{\prime }}=1,\;\;\;\;\;\beta_{i,i}^{t,t^{\prime }}=0\;\;\;\;\;\text{for}\;\;\text{all}\;i\geq 1,\;\;\;\;\;\beta_{0,j}^{t,t^{\prime }}=0\;\;\;\;\;\text{for}\;\;\text{all}\;j\geq 1,\;\;\;\;\;\beta_{i,j}^{t,t^{\prime }}=0\;\;\;\;\;\text{for}\;\;\text{all}\;i>j.$$

Consequently, one obtains

$$B_{0}=\beta_{0,0}^{t,t^{\prime }}=1,$$

and for each j≥1, the alternating sum simplifies to

$$B_{j}=\sum_{i=1}^{j-1}{\left(-1\right)}^{i}\beta_{i,j}^{t,t^{\prime }}.$$

### 4.2 k-mer algebraic representations of sequences

In this section, we review the k-mer representation framework introduced by Hozumi et al. [30], which provides a foundational method for embedding sequences as collections of integer sequences in a geometric space. Let A be a finite alphabet and let k>0 be an integer. A k-mer over A is a word $\text{x}=x_{1}x_{2}\cdots x_{k}\in A^{k}$. Given a fixed k-mer $\text{x}\in A^{k}$, we define the k-mer indicator function $\delta_{\text{x}}:A^{k}\to \left\{0,1\right\}$ by

(17) $$\delta_{\text{x}}\left(\text{y}\right)=\left\{\begin{array}{ll} 1, & \text{if}\;\;\text{y}=\text{x},\\ 0, & \text{otherwise}. \end{array}\right.$$

Given a sequence $S=s_{1}s_{2}\cdots s_{N}\in A^{N}$, we define the set of positions at which the k-mer x occurs in S as

(18) $$S^{\text{x}}=\left\{i\in \left[1,N-k+1\right]\left|\delta_{\text{x}}\left(s_{i}s_{i+1}\cdots s_{i+k-1}\right)=1\right.\right\}.$$

The corresponding pairwise distance matrix $D^{\text{x}}=\left(d_{ij}^{\text{x}}\left|i,j\in S^{\text{x}}\right.\right)$ is defined by

(19) $$d_{ij}^{\text{x}}=\left|i-j\right|,\;\;\;\;\;\;\;\;\;\;\text{for}\;\;\text{all}\;\;i,j\in S^{\text{x}}.$$

These distance matrices serve as the input for persistent Stanley–Reisner computations. Specifically, for each k-mer $\text{x}\in A^{k}$, the corresponding sequence of integers $S^{\text{x}}\subset \mathbb{R}$ gives rise to a family of Stanley–Reisner algebraic feature vectors computed over a filtration interval $\left[r_{0},r_{1}\right]$. For filtration values r, $r^{\prime }\in \left[r_{0},r_{1}\right]$ with $r\leq r^{\prime }$, we define

$$v_{\text{x}}^{r,r^{\prime }}={\left(v_{i}^{r,r^{\prime }}\left(\text{x}\right)\right)}_{i\in \mathbb{N}},$$

where $v_{i}^{r,r^{\prime }}\left(\text{x}\right)$ denotes a persistent invariant of dimension i, such as the f-vector, h-vector, or facet number, associated with the Vietoris–Rips complex built from $S^{\text{x}}$.

To simplify notation, we restrict to the diagonal case $r=r^{\prime }$, and denote the resulting feature vector by

$$v_{\text{x}}:={\left(v_{i}\left(\text{x}\right)\right)}_{i\in \mathbb{N}}.$$

For a fixed integer k>0, the full representation of the sequence $S\in A^{N}$ is given by the concatenation of these vectors over all k-mers:

$$\begin{array}{l} {\text{v}}_{S}^{k}:=\left(v_{\text{x}}\left|\text{x}\in A^{k}\right.\right)\\ \;\;\;={\left(v_{i}\left(\text{x}\right)\left|\text{x}\in A^{k}\right.\right)}_{i\in \mathbb{N}}, \end{array}$$

which we refer to as the k-mer algeraic representation of S at level k. This construction yields a feature vector indexed jointly by algebraic dimension i and k-mer $\text{x}\in A^{k}$.

### 4.3 Algebraic genetic distances

To compare two sequences $S_{1}\in A^{N_{1}}$ and $S_{2}\in A^{N_{2}}$, we define a family of weighted Euclidean metrics that aggregate Stanley–Reisner algebraic information across both the algebraic dimensions and k-mer lengths. Let $a_{k,i}\geq 0$ denote a non-negative weight assigned to homological dimension i at scale k. The dimension- and scale-weighted algebraic distance is defined by

(20) $$d_{v}\left(S_{1},S_{2}\right):=\sum_{k=1}^{K}\sum_{i=0}^{D_{k}}a_{k,i}\cdot {\left\|{\text{v}}_{S_{1},i}^{k}-{\text{v}}_{S_{2},i}^{k}\right\|}_{2},$$

where ${\text{v}}_{S,i}^{k}:=\left(v_{i}\left(\text{x}\right)\left|\text{x}\in A^{k}\right.\right)$ is the vector of dimension-i features computed over all k-mers in S, and $D_{k}$ is the maximum dimension considered for k. There are various strategies for selecting the weights $a_{k,i}$. One approach commonly found in the literature is to set $a_{k,i}=1/2^{k-1}$. To account for the influence of the dimension i, an alternative is to define $a_{k,i}=1/2^{i\cdot K+k-1}$, where K denotes the maximum window of k-mer lengths considered.

Within the CAKL framework, three distinct types of persistent algebraic features are employed to define pairwise distances between sequences. Specifically, the distance $d_{f}\left(S_{1},S_{2}\right)$ is derived from the f-vector curves, where the feature vector v is computed from these curves; the distance $d_{h}\left(S_{1},S_{2}\right)$ is defined analogously using h-vector curves; and the distance $d_{F}\left(S_{1},S_{2}\right)$ is based on the facet count vectors associated with the underlying filtration.

The final composite distance, integrating these three feature types, is given by

(21) $$d\left(S_{1},S_{2}\right):=d_{f}\left(S_{1},S_{2}\right)+d_{h}\left(S_{1},S_{2}\right)+d_{F}\left(S_{1},S_{2}\right),$$

which captures a broad range of persistent characteristics across multiple features and filtration levels. This composite metric constitutes the core of the CAKL approach to alignment-free sequence comparison.

In the applications considered in this work, we restrict our attention to a single type of feature representation—namely, the facet vector curves—and employ a fixed window length k for k-mers.

### 4.4 Computational simplicifications of the persistent h-vectors and f-vectors

Vietoris–Rips simplicial complexes arising from the k-mer algebraic representations possess a structural property that significantly simplifies their algebraic analysis. Specifically, many of the persistent graded Betti numbers vanish in higher homological degrees, which reduces the complexity of computations involving persistent h-vectors. The following proposition formalizes this observation and highlights its relevance to k-mer algebraic representations:

#### Proposition 4.1.

Let $\Delta =\Delta^{t}$ denote the Vietoris–Rips complex at scale t associated with a sequence X⊂ℝ. Then for every subset W⊆V and every j≥2, the persistent Betti numbers satisfy

$$\beta_{j-1}^{t,t^{\prime }}\left(\Delta_{W}\right)=0.$$

As a consequence,

$$\beta_{i,i+j}^{t,t^{\prime }}=0\;\;\;\;for\;\;all\;\;i\geq 1,\;\;j\geq 2,$$

and the only potentially nonzero contributions occur in degree shifts of one, namely

$$\beta_{i,i+1}^{t,t^{\prime }}=\sum_{\begin{array}{l} \;\;\;\;W\subseteq V\\ \left|W\right|=i+1 \end{array}}\left(\beta_{0}^{t,t^{\prime }}\left(\Delta_{W}\right)-1\right).$$

Therefore, the alternating sum of persistent Betti numbers at total degree j simplifies to

$$B_{j}:=\sum_{i=0}^{j}{\left(-1\right)}^{i}\beta_{i,j}^{t,t^{\prime }}={\left(-1\right)}^{j-1}\beta_{j-1,j}^{t,t^{\prime }}\;\;\;for\;\;all\;\;j\geq 1.$$

In particular, the persistent h-vector expression in equation (16) becomes

$$h_{m}^{t,t^{\prime }}=\alpha_{0}^{\left(m\right)}+\sum_{j=1}^{m}\alpha_{j}^{\left(m\right)}{\left(-1\right)}^{j-1}\beta_{j-1,j}^{t,t^{\prime }},\;\;\;\;\;with\;\;h_{0}^{t,t^{\prime }}=1.$$

To establish Proposition 4.1, we prove a more general structural result concerning Vietoris–Rips complexes over sequences in ℝ. Let X⊆ℝ be a finite sequence, and let ${\text{VR}}_{\epsilon }\left(X\right)$ denote the Vietoris–Rips complex at scale ϵ.

Each facet $F\subseteq {\text{VR}}_{\epsilon }\left(X\right)$ admits a unique minimal element $x=\inf\left(F\right)\in X$. Moreover, if another facet $G\subseteq {\text{VR}}_{\epsilon }\left(X\right)$ satisfies $\inf\left(G\right)=x$, then necessarily G=F. That is, the minimal element uniquely determines the facet. Consequently, the assignment $x\mapsto F_{x}$, where $F_{x}$ denotes the unique facet with minimal element x, satisfies

$$F_{x}=F_{y}\Leftrightarrow x=y.$$

In particular, the collection of facets is in bijective correspondence with the set of the minimal elements of the facets, and hence can be linearly ordered by their infima:

$$F_{x}\leq F_{y}\Leftrightarrow x\leq y.$$

#### Proposition 4.2.

Let X⊆ℝ be a finite sequence. Then for all q≥1,

$$H_{q}\left({\text{VR}}_{\epsilon }\left(X\right)\right)=0.$$

*Proof.* Let ${\text{VR}}_{\epsilon }\left(X\right)=\cup_{i=1}^{n}F_{x_{i}}$, where the facets $F_{x_{i}}$ are ordered such that $x_{1}<x_{2}<\cdots <x_{n}$.

We proceed by induction on n.

Base case: When n=1, ${\text{VR}}_{\epsilon }\left(X\right)$ is a single simplex, which is contractible. Therefore, $H_{q}=0$ for all q≥1.

Inductive step: Assume the result holds for n−1 facets, where n>1. Let:

$$K_{1}=\overset{n-1}{\underset{i=1}{\cup }}F_{x_{i}},\;\;\;\;\;K_{2}=F_{x_{n}},\;\;\;\;\;K=K_{1}\cup K_{2}.$$

Note that $K_{1}$, $K_{2}$, and $K_{1}\cap K_{2}$ are all simplicial complexes. Notice that the vertices of $K_{1}\cap K_{2}$ lie within the interval $\left[x_{n},x_{n-1}+\epsilon \right]$, whose length is at most ϵ, the intersection $K_{1}\cap K_{2}$ is either empty or a simplex.

By the Mayer–Vietoris sequence, we obtain the long exact sequence in homology:

$$\cdots \to H_{q}\left(K_{1}\cap K_{2}\right)\to H_{q}\left(K_{1}\right)⊕H_{q}\left(K_{2}\right)\to H_{q}\left(K\right)\to H_{q-1}\left(K_{1}\cap K_{2}\right)\to \cdots$$

By the inductive hypothesis, $H_{q}\left(K_{1}\right)=0$ for all q≥1, and since $K_{2}$ is a simplex, $H_{q}\left(K_{2}\right)=0$ as well. Furthermore, $K_{1}\cap K_{2}$ is either a simplex or empty, so:

$$H_{q}\left(K_{1}\cap K_{2}\right)=0\;\;\;\text{for}\;\;\text{all}\;\;q\geq 1.$$

Thus, the exact sequence reduces to:

$$0\to H_{q}\left(K\right)\to 0\Rightarrow H_{q}\left(K\right)=0\;\;\;\text{for}\;\;\text{all}\;\;q\geq 2.$$

To analyze $H_{1}\left(K\right)$, we consider:

$$0\to H_{1}\left(K\right)\to H_{0}\left(K_{1}\cap K_{2}\right)\to H_{0}\left(K_{1}\right)⊕H_{0}\left(K_{2}\right)\to H_{0}\left(K\right)\to 0.$$

If $K_{1}\cap K_{2}=\emptyset$, then $H_{1}\left(K\right)=0$ since $K=K_{1}⊔K_{2}$ is a disjoint union of two contractible subcomplexes. Thus, the result holds in this case.

Suppose instead that $K_{1}\cap K_{2}\neq \emptyset$. Then $K_{1}\cap K_{2}$ is a simplex and hence contractible, in particular connected. Since $K_{2}$ is also a simplex, it is connected and contractible. Moreover, the inclusion of $K_{2}$ into $K=K_{1}\cup K_{2}$ does not change the number of connected components, so K and $K_{1}$ have the same number of components. Therefore, the canonical map

$$H_{0}\left(K_{1}\right)⊕H_{0}\left(K_{2}\right)\to H_{0}\left(K\right)$$

has kernel of dimension one, namely $\dim H_{0}\left(K_{2}\right)=1$. Since $K_{1}\cap K_{2}$ is connected, the induced map

$$H_{0}\left(K_{1}\cap K_{2}\right)\to H_{0}\left(K_{1}\right)⊕H_{0}\left(K_{2}\right)$$

is injective. Consequently, in the Mayer–Vietoris sequence, the connecting homomorphism

$$H_{1}\left(K\right)\to H_{0}\left(K_{1}\cap K_{2}\right)$$

must be the zero map. It follows that $H_{1}\left(K\right)=0$ in this case as well. By induction on the number of simplices, we conclude that

$$H_{q}\left({\text{VR}}_{\epsilon }\left(X\right)\right)=0\;\;\;\text{for}\;\;\text{all}\;\;q\geq 1.$$

This structural property does not extend to higher-dimensional ambient spaces $X\subset {\mathbb{R}}^{d}$ for d≥2; for instance, consider the Vietoris–Rips complex formed from the vertices of a regular hexagon in ${\mathbb{R}}^{2}$. Therefore, Proposition 4.1 is a consequence of the special linear ordering available in one-dimensional point clouds. This leads to a significant simplification in the computation of persistent Betti numbers arising from k-mer algebraic representations.

Given a simplicial complex Δ, its 1-skeleton induces an undirected graph $G\left(\Delta \right)$ with vertex set V and edge set

$$E\left(\Delta \right):=\left\{\left\{v_{i},v_{j}\right\}\subseteq V\left|\left\{v_{i},v_{j}\right\}\right.\in \Delta \right\}.$$

When Δ is a Vietoris–Rips complex built on k-mer represetations in ℝ, the persistent Betti numbers of Δ are entirely determined by the topology of the associated graph $G\left(\Delta \right)$. This follows directly from Proposition 4.1. We formalize this relationship in the following theorem:

#### Theorem 4.3.

Let Δ be a Vietoris–Rips simplicial complex on a finite sequence X⊂ℝ, and let $G\left(\Delta \right)=\left(V,E\left(\Delta \right)\right)$ denote its 1-skeleton. Then the persistent Betti numbers of Δ satisfy:

$$\beta_{i,i+1}\left(G\left(\Delta \right)\right)=\beta_{i,i+1}\left(\Delta \right),\;\;\;\;\;and\;\;\;\;\;\beta_{i,i+j}\left(G\left(\Delta \right)\right)=\beta_{i,i+j}\left(\Delta \right)=0\;\;\;\;\;for\;\;all\;\;j\geq 2.$$

### 4.5 Purity metrics for assessing the performance of phylogenetic analysis methods

We introduce a purity metrics for assessing monophyly in phylogenetic trees. Let S be a finite set of size $n=\left|S\right|$, and let $P=\left\{S_{1},S_{2},\ldots ,S_{k}\right\}$ be a partition of S into disjoint subsets such that $\cup_{i=1}^{k}S_{i}=S$. We define the *purity* of the partition P as

(22) $$\text{purity}\left(P\right)={\sum_{i=1}^{k}\left(\frac{\left|S_{i}\right|}{n}\right)}^{2}.$$

This quantity reflects the degree to which elements are concentrated within the subsets of the partition. A higher purity indicates that the majority of elements reside in a small number of large subsets, while a lower purity corresponds to a more evenly distributed partition.

To illustrate, consider several representative scenarios. If the partition is perfect in the sense that all elements are grouped into a single subset, i.e., k=1, then

$$\text{purity}\left(P\right)={\left(\frac{n}{n}\right)}^{2}=1,$$

which is the maximal possible value. If the partition consists of n singleton subsets (i.e., $\left|S_{i}\right|=1$ for all i), then

$$\text{purity}\left(P\right)={\sum_{i=1}^{n}\left(\frac{1}{n}\right)}^{2}=\frac{1}{n},$$

which is minimal. If the partition consists of two equal-sized subsets, each of size n/2, then

$$\text{purity}\left(P\right)=2{\left(\frac{1}{2}\right)}^{2}=\frac{1}{2}.$$

Finally, if one subset dominates the partition, for example, with $\left|S_{1}\right|=n-1$ and $\left|S_{2}\right|=1$, then

$$\text{purity}\left(P\right)={\left(\frac{n-1}{n}\right)}^{2}+{\left(\frac{1}{n}\right)}^{2}=1-\frac{2\left(n-1\right)}{n^{2}},$$

which approaches 1 as n→∞, but is strictly less than 1 for any finite n.

Let S be a finite set of leaf nodes in a phylogenetic tree, and let each element of S be assigned a categorical label (e.g., species, clade, or functional class). For each label ℓ, let $S^{\left(\ell \right)}\subseteq S$ denote the set of leaves with label ℓ, and let $n^{\left(\ell \right)}=\left|S^{\left(\ell \right)}\right|$ be the number of such leaves.

To assess the purity of the tree with respect to label ℓ, we identify all maximal subtrees whose leaves are exclusively labeled ℓ. These subtrees define a partition $P_{\ell }=\left\{S_{1},S_{2},\ldots ,S_{k}\right\}$ of $S^{\left(\ell \right)}$, where each $S_{i}\subseteq S^{\left(\ell \right)}$ is the set of leaves in a pure subtree.

The purity of the label ℓ is then defined as:

(23) $$\text{purity}\left(P_{\ell }\right)={\sum_{i=1}^{k}\left(\frac{\left|S_{i}\right|}{n^{\left(\ell \right)}}\right)}^{2},$$

where the numerator $\left|S_{i}\right|$ denotes the size of a pure subtree and the denominator normalizes by the total number of leaves of label ℓ.

A purity of 1.0 indicates that all leaves of label ℓ are perfectly clustered under a single subtree (i.e., monophyletic), while a lower purity reflects fragmentation of that label across multiple subtrees. Averaging the purity scores across all labels provides an overall measure of the taxonomic coherence of the tree:

(24) $$\text{avg\_purity}=\frac{1}{\left|L\right|}\sum_{\ell \in L}\text{purity}\left(P_{\ell }\right),$$

where L is the set of all unique labels in the tree.

This approach is beneficial for evaluating the extent to which a phylogenetic tree respects known groupings, such as taxonomic families or functional clusters, without requiring an explicit reference.

## Supplementary Material

1

Supplementary materials are available for performance comparison of different methods on phylogenetic analysis.

## Figures and Tables

**Table 1:** Comparison of 1-NN classification accuracies of the six methods, where the scores of NVM, FFP-JSm FFP-KL, FPS and Markov are obtained from [30].

Data	CAKL	NVM	FPS-JS	FFP-KL	FPS	Markov
NCBI 2020	**0.932**	0.879	0.862	0.862	0.732	0.734
NCBI 2022	**0.920**	0.875	0.870	0.870	0.732	0.735
NCBI 2024	**0.891**	0.829	0.825	0.826	0.656	0.637
NCBI 2024 All	**0.892**	0.825	0.832	0.832	0.647	0.647

## Data Availability

The implementation of the proposed CAKL framework is available at https://github.com/FaisalSuwayyid/dlCAKL including the source code for the methods used for comparison in this study.
