# Supplementary material for: CAKL: Commutative algebra k-mer learning of genomics
Source: ArXiv. 2025 Aug 13:arXiv:2508.09406v1. Preprint. [Version 1] (PMC12364056)
Supplement: 1 [file NIHPP2508.09406v1-supplement-1.pdf]

Supplementary Information

S1 Datasets

To rigorously evaluate the proposed method CAKL, we assembled a suite of benchmark datasets spanning diverse applications in genomics and virology.

Genetic Variant Identification. To evaluate the discriminatory capacity of CAKL in distinguishing between genetic variants, we employed the SARS-CoV-2 dataset curated by Hozumi *et al.* [30], which comprises representative genome sequences from multiple SARS-CoV-2 lineages. The dataset consists of 44 complete genomes of SARS-CoV-2, sourced from GISAID. These genomes are classified according to their variant lineages, including Alpha, Beta, Gamma, Delta, Lambda, Mu, GH/490R, and Omicron. Phylogenetic branches and labels are annotated and color-coded to reflect these variant classifications.

Phylogenetic Reconstruction. Six benchmark genome collections were employed for phylogenetic tree construction. These datasets, compiled in early studies and detailed in [30], span a range of evolutionary scales and biological taxa, providing a robust framework for assessing the accuracy of phylogenetic inference.

The datasets span a broad range of sequence lengths. For example, Influenza A hemagglutinin (HA) genes consist of approximately 2,000 nucleotides; human rhinovirus (HRV) genomes and hepatitis E viruses (HEV) range around 7,000 nucleotides; mammalian mitochondrial genomes and Ebola virus (EBOV) genomes contain roughly 17,000 nucleotides; and bacterial genomes typically range from several hundred thousand to a few million nucleotides.

The mammalian mitochondrial dataset comprises 41 species across several mammalian orders: Primates, Carnivora, and Cetacea from Euarchontoglires, and Artiodactyla, Perissodactyla, Lagomorpha, Rodentia, and Erinaceomorpha from Laurasiatheria. The objective is to evaluate the extent to which each method reconstructs clades consistent with established host species classifications.

The HRV dataset comprises 113 complete HRV genomes consisting of three main groups, HRV-A, HRV-B, and HRV-C, along with three outgroup sequences (HEV).

The HEV dataset comprises 48 complete genomes of HEV grouped into four major genotypic categories, Group 1, Group 2, Group 3 and Group 4.

The influenza HA genes dataset contains 30 Influenza A hemagglutinin (HA) genes classified into six well-characterized subtypes—H1N1, H2N2, H3N2, H5N1, H7N9, and H7N3.

Ebolavirus genomes dataset includes 59 complete genomes of Ebola virus categorized into five viral types: Bundibugyo virus (BDBV), Reston virus (RESTV), Ebola virus (EBOV), Sudan virus (SUDV), and Tai Forest virus (TAFV), where EBOV sequences are further annotated by epidemic location and year, enabling evaluation of phylogenetic resolution at both species and outbreak levels.

The bacterial genomes dataset comprises 30 complete bacterial genomes, classified into nine bacterial families: Bacillaceae, Borreliaceae, Burkholderiaceae, Clostridiaceae, Desulfovibrionaceae, Enterobacteriaceae, Rhodobacteraceae, Staphylococcaceae, and Yersiniaceae. The genome sizes of Borreliaceae range from approximately 0.9 to 2.5 Mb, whereas those of Enterobacteriaceae span 4.0 to 6.5 Mb.

Viral Family Classification. For viral classification tasks, we adopted four datasets derived from the NCBI Virus Database (`https://www.ncbi.nlm.nih.gov/labs/virus/vssi/`), each annotated with taxonomic labels at the viral family level. These datasets include:

1. **NCBI 2020**: Contains 6,993 viral genomes and was originally collected in Sun *et al.* [31];
2. **NCBI 2022**: Comprises 11,428 genomes, as used in Yu *et al.* [27];
3. **NCBI 2024**: A refined version of the NCBI All dataset, from which entries lacking the "-viridae" suffix and sequences containing invalid nucleotides were removed as in [30].
4. **NCBI 2024 All**: Includes 13,645 genomes collected by Hozumi *et al.* as of January 20, 2024 [30];

Reference genomes were obtained directly from the NCBI Virus database, with viral family labels defined according to the taxonomy of the International Committee on Taxonomy of Viruses (ICTV). It is important to note that the NCBI database undergoes continual curation. Consequently, several reference sequences used in prior studies are no longer available and were excluded from our analysis, following the filtering strategy of [30]. Additionally, certain viral sequences have been reassigned to updated taxonomic lineages. For consistency and comparability, we retained the original lineage assignments as reported in the source publications [30, 27, 31].

To ensure sufficient representation within each taxonomic class, viral families represented by a single reference genome were excluded from all datasets. A comprehensive overview of dataset composition, including filtering criteria and collection metadata, is presented in Table S1. For further methodological details on dataset construction and curation, we refer the reader to [30, 27, 31].

S2 Viral variant identification

Fig. S1 shows the phylogenetic trees inferred by six alignment-free methods on the SARS-CoV-2 dataset used for genetic variant identification. Among these methods, our CAKL approach achieved the highest concordance with known variant lineages, outperforming the other five. Compared with the MAFFT-based tree (Fig. S8), which serves as a state-of-the-art alignment-based benchmark, the CAKL tree captures the same high-level clade structure and accurately delineates all major SARS-CoV-2 lineages. While subtle differences in internal branching order exist, both trees identify consistent and biologically meaningful variant groupings.

Notably, the CAKL tree closely mirrors the MAFFT tree in its high-level topology, despite being derived entirely without the use of sequence alignment. This highlights the strength of CAKL as a reliable and scalable alignment-free approach to phylogenetic inference. Its ability to reproduce biologically meaningful relationships among viral variants reinforces its potential for large-scale genomic studies where alignment may be computationally prohibitive or error-prone.

S3 Phylogenetic analysis

This section presents the phylogenetic trees generated by the six methods evaluated in this study across the six datasets used for phylogenetic analysis. Among these methods, CAKL demonstrated

**Dataset (Reference)**	**Date**	**#Fam.**	**#Seq.**	**Preprocessing Criteria**
NCBI 2020 [31]	Mar 2020	83	6,993	Unknown Baltimore class<br>Unknown family<br>Families with <2 sequences
NCBI 2022 [27]	Mar 2022	123	11,428	Partial sequences<br>Unknown family<br>Families with <2 sequences<br>Invalid nucleotides
NCBI 2024 [30] (Filtered)	Jan, 2024	199	12,154	Partial sequences<br>Unknown family<br>Only "-viridae"<br>Families with <2 sequences<br>Invalid nucleotides
NCBI 2024 [30] (All)	Jan, 2024	209	13,645	Partial sequences<br>Unknown family<br>Families with <2 sequences

Table S1: Summary of NCBI viral genome datasets, including collection date, preprocessing steps, number of families, and number of sequences [30].

consistently stable performance across all datasets. In contrast, the other methods showed varying performance depending on the dataset, highlighting their sensitivity to data characteristics.

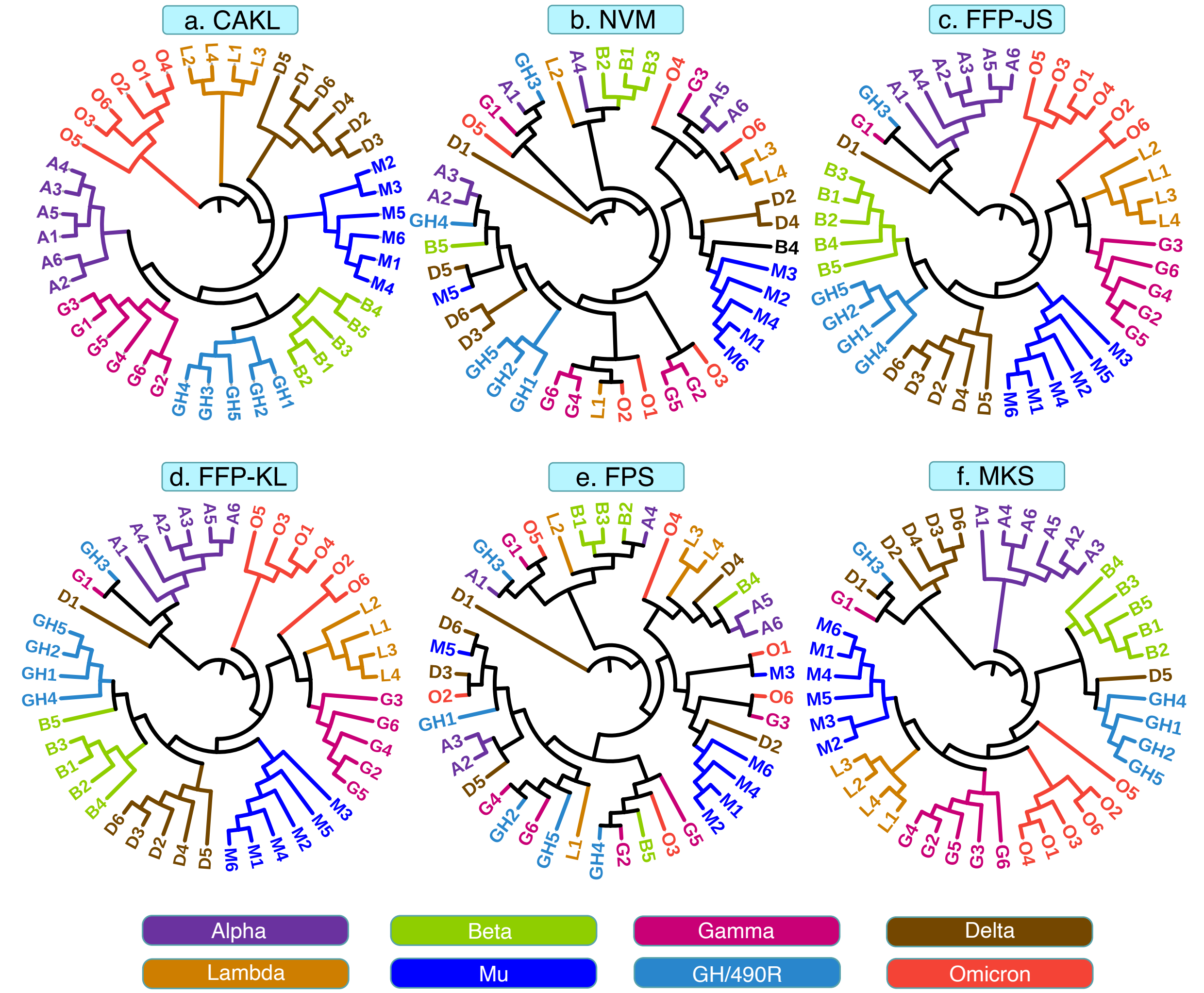

Figure S1: Performance comparison of various methods for SARS-CoV-2 variant identification was conducted on a dataset comprising 44 complete genomes of severe acute respiratory syndrome coronavirus 2 (SARS-CoV-2), sourced from the GISAID database. CAKL accurately grouped all variant sequences. NVM revealed minimal structure. FFP-KL and FFP-JS misclassified three genomes; Markov misclassified three as well; FPS produced no discernible clustering.

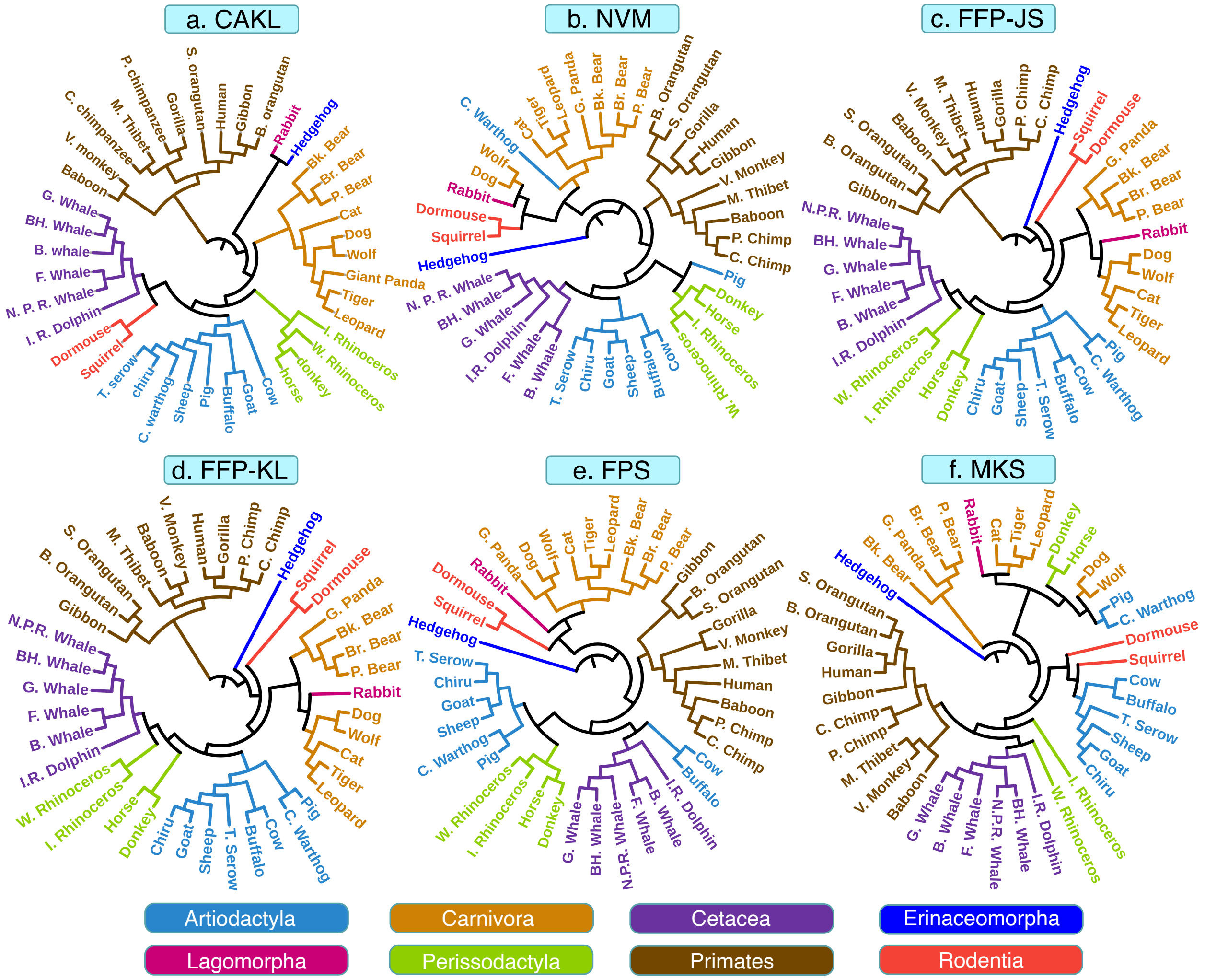

Figure S2: Performance comparison of various methods on the dataset of 42 complete mammalian mitochondrial genomes. The CAKL method accurately clustered all sequences by their known taxonomy. NVM failed to group warthog and pig with *Artiodactyla*, and produced fragmented *Carnivora* clades. Both FFP-JS and FFP-KL separated *Carnivora* and *Perissodactyla* into multiple clades. The Markov method resulted in three distinct *Carnivora* clades, failed to form coherent clusters for *Rodentia* and *Perissodactyla*, and fragmented Artiodactyla. The FPS method split *Artiodactyla* into two separate clades.

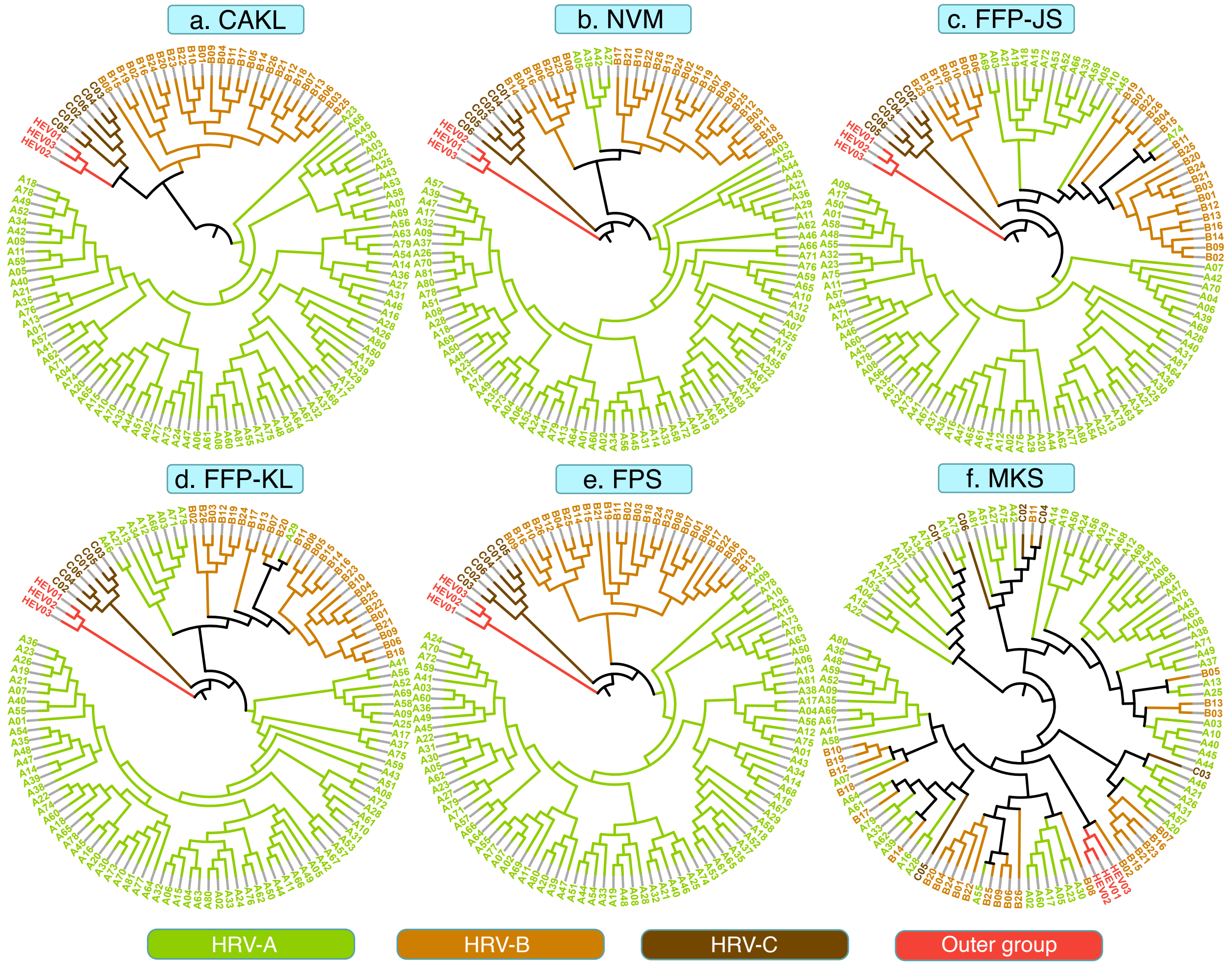

Figure S3: Performance comparison of various methods was carried out on a dataset comprising 113 complete genomes of human rhinoviruses (HRVs), supplemented with three outgroup sequences from the HEV. CAKL and FPS correctly grouped all HRV genomes and separated them from outgroup sequences. NVM, FFP-JS, and FFP-KL each misclassified one or more HRV-A genomes within the HRV-B clade. Markov failed to produce uniform HRV clades and did not separate the outgroups.

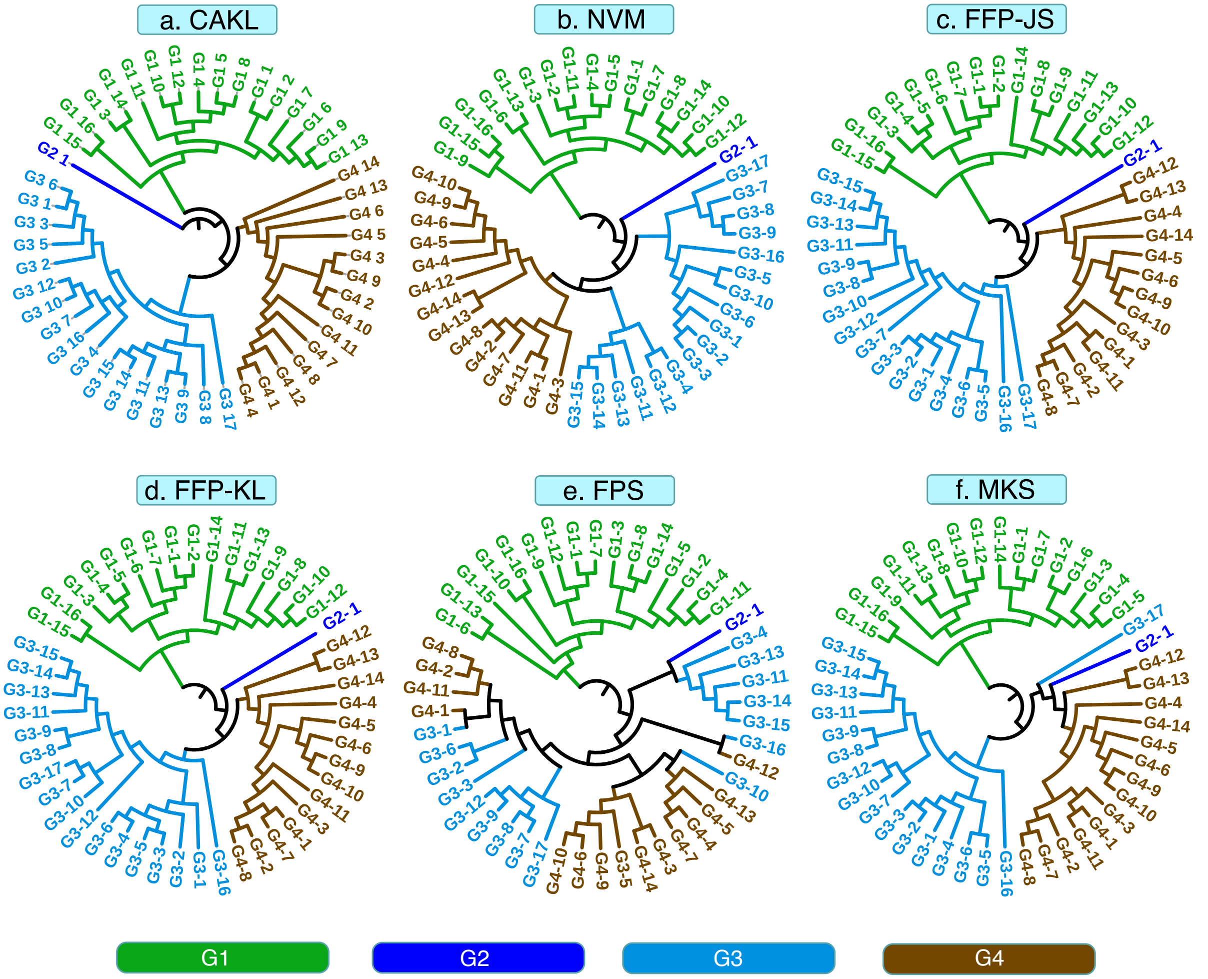

Figure S4: Performance comparison of various methods on the dataset of 48 complete Hepatitis E virus genomes (HEV). CAKL, FFP-JS, FFP-KL, and NVM correctly grouped all sequences. Markov misclassified one Group 3 genome, and FPS did not separate Groups 3 and 4.

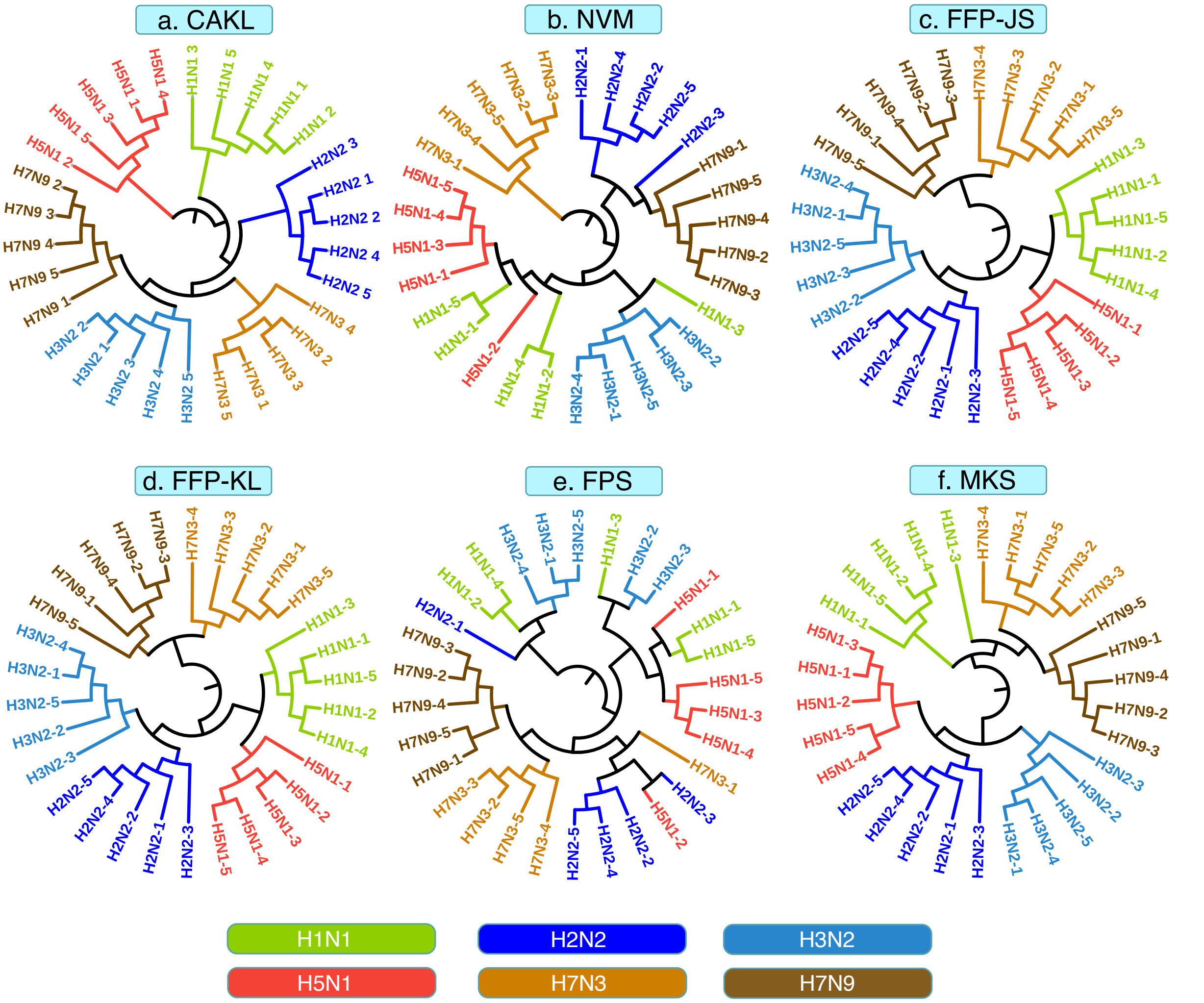

Figure S5: Performance comparison of various methods on the dataset of 30 influenza HA genes. CAKL, FFP-JS, and FFP-KL formed all clades correctly. NVM failed to group all H1N1 sequences and misclassified one H2N2 sequence; Markov misclassified one H1N1 gene. FPS did not produce clear clustering for most subtypes.

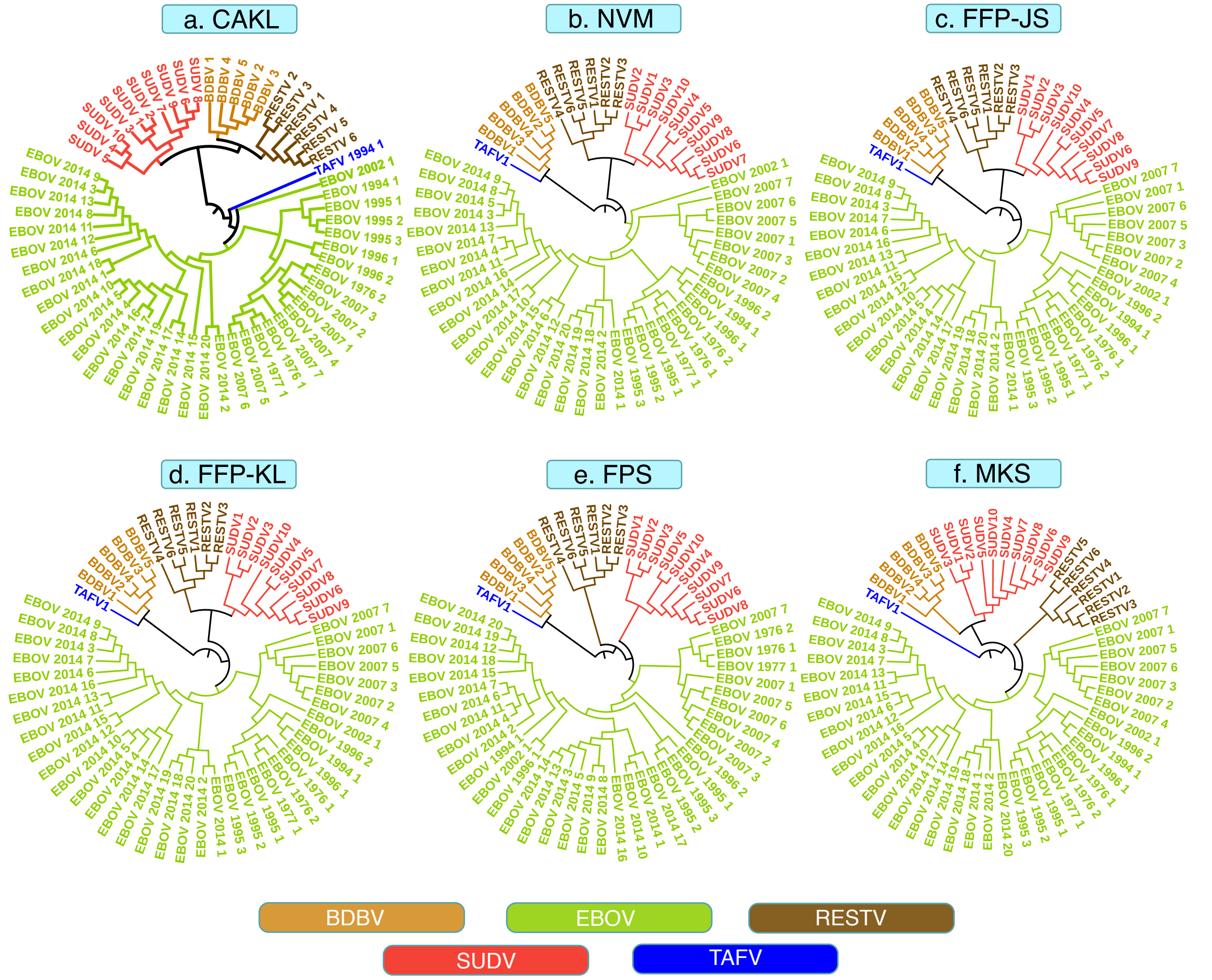

Figure S6: Performance comparison of various methods on the dataset of 59 complete genomes of ebolaviruses. All methods correctly recovered the viral types. Markov yielded a tree in which EBOV and RESTV shared a node, and FPS did not cluster EBOV epidemic strains distinctly.

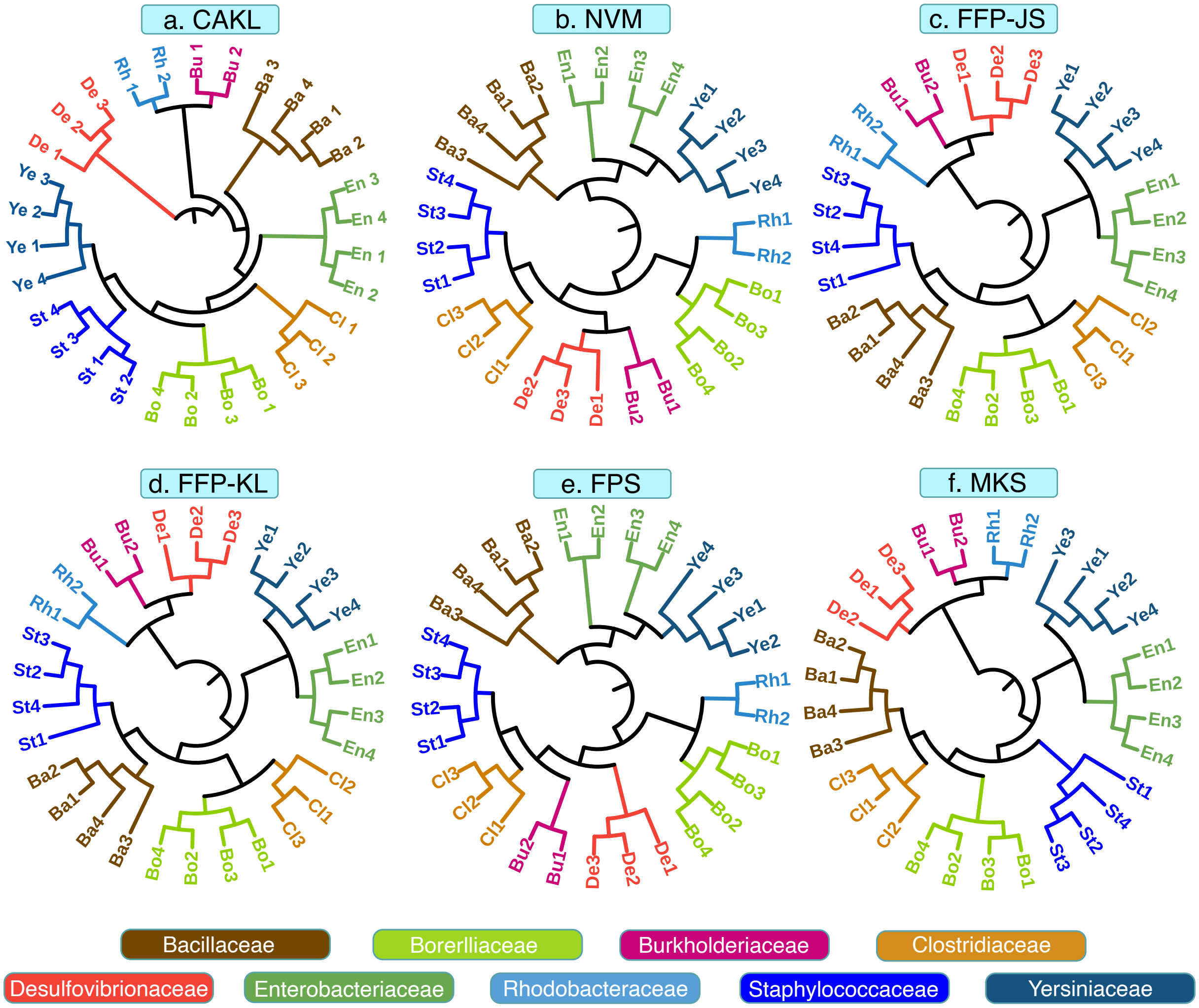

Figure S7: Performance comparison of various methods on a dataset of 30 complete bacterial genomes revealed that all methods—except NVM and FPS, which split the Enterobacteriaceae clade—successfully recovered the known taxonomic groupings without error.

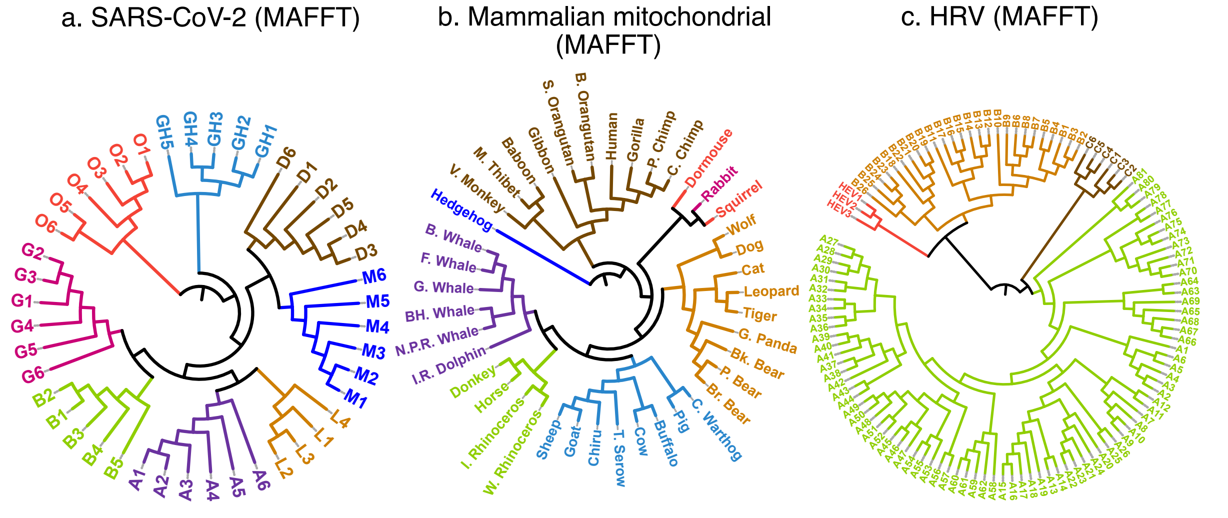

Figure S8: The phylogenetic trees constructed for the SARS-CoV-2, mammalian mitochondrial genomes, and human rhinovirus (HRV) datasets using MAFFT—an alignment-based method—exhibit a high degree of similarity to the corresponding trees generated by our proposed CAKL framework. This concordance underscores the biological validity of the alignment-free representations produced by CAKL.

S4 Viral classification

As discussed early, four datasets for viral classification were taken from [30]. A detailed comparison of CAKL and other five methods on classification tasks is given in Table S2.

Data	Method	ACC	BA	F1	Recall	Precision
NCBI 2020	CAKL	**0.913**	**0.887**	**0.892**	**0.887**	**0.915**
	NVM	0.847	0.807	0.809	0.807	0.840
	FFP-JS	0.821	0.790	0.781	0.790	0.814
	FFP-KL	0.819	0.789	0.780	0.789	0.814
	Markov	0.713	0.644	0.622	0.644	0.668
	FPS	0.714	0.637	0.633	0.637	0.665
NCBI 2022	CAKL	**0.902**	**0.820**	**0.824**	**0.819**	**0.859**
	NVM	0.852	0.747	0.750	0.747	0.791
	FFP-JS	0.830	0.740	0.733	0.740	0.769
	FFP-KL	0.832	0.743	0.735	0.743	0.771
	Markov	0.724	0.593	0.577	0.593	0.617
	FPS	0.723	0.588	0.580	0.588	0.603
NCBI 2024	CAKL	**0.876**	**0.792**	**0.804**	**0.792**	**0.853**
	NVM	0.814	0.729	0.738	0.729	0.789
	FFP-JS	0.796	0.724	0.712	0.724	0.744
	FFP-KL	0.796	0.727	0.714	0.727	0.747
	Markov	0.633	0.589	0.554	0.589	0.573
	FPS	0.660	0.561	0.560	0.561	0.593
NCBI 2024 All	CAKL	**0.876**	**0.794**	**0.807**	**0.794**	**0.853**
	NVM	0.809	0.729	0.738	0.729	0.788
	FFP-JS	0.799	0.721	0.711	0.721	0.745
	FFP-KL	0.799	0.724	0.712	0.724	0.745
	Markov	0.638	0.589	0.555	0.589	0.575
	FPS	0.651	0.561	0.559	0.561	0.591

Table S2: Comparison of 5-NN classification scores of the six methods, where the scores of NVM, FFP-JSm FFP-KL, Markov and FPS are obtained from [30].

References

[1] Gerald M Rubin, Mark D Yandell, Jennifer R Wortman, George L Gabor, Miklos, Catherine R Nelson, Iswar K Hariharan, Mark E Fortini, Peter W Li, Rolf Apweiler, et al. Comparative genomics of the eukaryotes. *Science*, 287(5461):2204–2215, 2000.

[2] Kelly A Frazer, Lior Pachter, Alexander Poliakov, Edward M Rubin, and Inna Dubchak. VISTA: computational tools for comparative genomics. *Nucleic acids research*, 32(suppl_2):W273–W279, 2004.

[3] Masatoshi Nei. Phylogenetic analysis in molecular evolutionary genetics. *Annual Review of Genetics*, 30(1):371–403, 1996.

[4] Matthew I. Bellgard, Takeshi Itoh, Hidemi Watanabe, Tadashi Imanishi, and Takashi Gojobori. Dynamic evolution of genomes and the concept of genome space. *Annals of the New York Academy of Sciences*, 870(1):293–300, 1999.

[5] Fabian Sievers, Andreas Wilm, David Dineen, Toby J. Gibson, Kevin Karplus, Weizhong Li, Rodrigo Lopez, Hamish McWilliam, Michael Remmert, Johannes Söding, and et al. Fast, scalable generation of high-quality protein multiple sequence alignments using clustal omega. *Molecular Systems Biology*, 7(1):539, 2011.

[6] Kazutaka Katoh and Daron M. Standley. Mafft multiple sequence alignment software version 7: improvements in performance and usability. *Molecular Biology and Evolution*, 30(4):772–780, 2013.

[7] Robert C. Edgar. Muscle: multiple sequence alignment with high accuracy and high throughput. *Nucleic Acids Research*, 32(5):1792–1797, 2004.

[8] Michael Bleher, Lukas Hahn, Maximilian Neumann, Juan Angel Patino-Galindo, Mathieu Carriere, Ulrich Bauer, Raul Rabadan, and Andreas Ott. Topological data analysis identifies emerging adaptive mutations in sars-cov-2. *arXiv preprint arXiv:2106.07292*, 2021.

[9] Juan Ángel Patiño-Galindo, Ioan Filip, Ratul Chowdhury, Costas D. Maranas, Peter K. Sorger, Mohammed AlQuraishi, and Raul Rabadan. Recombination and lineage-specific mutations linked to the emergence of sars-cov-2. *Genome Medicine*, 13(1):124, 2021.

[10] Susana Vinga. Editorial: Alignment-free methods in computational biology. *Briefings in Bioinformatics*, 15(3):341–342, 2014.

[11] Andrzej Zielezinski, Susana Vinga, Jonas Almeida, and Wojciech M. Karlowski. Alignment-free sequence comparison: benefits, applications, and tools. *Genome Biology*, 18:1–17, 2017.

[12] Oliver Bonham-Carter, Joe Steele, and Dhundy Bastola. Alignment-free genetic sequence comparisons: a review of recent approaches by word analysis. *Briefings in Bioinformatics*, 15(6):890–905, 2014.

[13] Guillaume Bernard, Cheong Xin Chan, and Mark A. Ragan. Alignment-free microbial phylogenomics under scenarios of sequence divergence, genome rearrangement and lateral genetic transfer. *Scientific Reports*, 6(1):28970, 2016.

[14] Andrzej Zielezinski, Hani Z. Girgis, Guillaume Bernard, Chris-Andre Leimeister, Kujin Tang, Thomas Dencker, Anna Katharina Lau, Sophie Röhling, Jae Jin Choi, Michael S. Waterman, Matteo Comin, Sung-Hou Kim, Susana Vinga, Jonas S. Almeida, Cheong Xin Chan, Benjamin T. James, Fengzhu Sun, Burkhard Morgenstern, Wojciech M. Karlowski, and Raul Rabadan. Benchmarking of alignment-free sequence comparison methods. *Genome Biology*, 20(1):144, 2019.

[15] B. Edwin Blaisdell. A measure of the similarity of sets of sequences not requiring sequence alignment. *Proceedings of the National Academy of Sciences*, 83(14):5155–5159, 1986.

[16] Myron Tribus and Edward C. McIrvine. Energy and information. *Scientific American*, 225(3):179–190, 1971.

[17] Hasan H. Otu and Khalid Sayood. A new sequence distance measure for phylogenetic tree construction. *Bioinformatics*, 19(16):2122–2130, 2003.

[18] Ming Li and Paul Vitányi. *An Introduction to Kolmogorov Complexity and Its Applications*, volume 3. Springer, 3rd edition, 2008.

[19] Chenglong Yu, Troy Hernandez, Hui Zheng, Shek-Chung Yau, Hsin-Hsiung Huang, Rong Lucy He, Jie Yang, and Stephen S.-T. Yau. Real time classification of viruses in 12 dimensions. *PloS One*, 8(5):e64328, 2013.

[20] Mo Deng, Chenglong Yu, Qian Liang, Rong L. He, and Stephen S.-T. Yau. A novel method of characterizing genetic sequences: genome space with biological distance and applications. *PloS One*, 6(3):e17293, 2011.

[21] H. Joel Jeffrey. Chaos game representation of gene structure. *Nucleic Acids Research*, 18(8):2163–2170, 1990.

[22] Milan Randić, Marjana Novič, and Dejan Plavšić. Milestones in graphical bioinformatics. *International Journal of Quantum Chemistry*, 113(22):2413–2446, 2013.

[23] Tung Hoang, Changchuan Yin, Hui Zheng, Chenglong Yu, Rong Lucy He, and Stephen S.-T. Yau. A new method to cluster dna sequences using fourier power spectrum. *Journal of Theoretical Biology*, 372:135–145, 2015.

[24] Changchuan Yin, Ying Chen, and Stephen S.-T. Yau. A measure of dna sequence similarity by fourier transform with applications on hierarchical clustering. *Journal of Theoretical Biology*, 359:18–28, 2014.

[25] Ajay Kumar Saw, Garima Raj, Manashi Das, Narayan Chandra Talukdar, Binod Chandra Tripathy, and Soumyadeep Nandi. Alignment-free method for dna sequence clustering using fuzzy integral similarity. *Scientific Reports*, 9(1):3753, 2019.

[26] Chenglong Yu, Qian Liang, Changchuan Yin, Rong L. He, and Stephen S.-T. Yau. A novel construction of genome space with biological geometry. *DNA Research*, 17(3):155–168, 2010.

[27] Hongyu Yu and Stephen S.-T. Yau. The optimal metric for viral genome space. *Computational and Structural Biotechnology Journal*, 23:2083–2096, 2024.

[28] Gregory E. Sims, Se-Ran Jun, Guohong A. Wu, and Sung-Hou Kim. Alignment-free genome comparison with feature frequency profiles (ffp) and optimal resolutions. *Proceedings of the National Academy of Sciences*, 106(8):2677–2682, 2009.

[29] Faisal Suwayyid and Guo-Wei Wei. Persistent stanley–reisner theory. *Foundations of Data Science*, page Doi: 10.3934/fods.2025009, 2025.

[30] Yuta Hozumi and Guo-Wei Wei. Revealing the shape of genome space via k-mer topology. *arXiv preprint arXiv:2412.20202*, 2024.

[31] Nan Sun, Shaojun Pei, Lily He, Changchuan Yin, Rong Lucy He, and Stephen S.-T. Yau. Geometric construction of viral genome space and its applications. *Computational and Structural Biotechnology Journal*, 19:4226–4234, 2021.

[32] Tiee-Jian Wu, Ya-Ching Hsieh, and Lung-An Li. Statistical measures of dna sequence dissimilarity under markov chain models of base composition. *Biometrics*, 57(2):441–448, 2001.

[33] Kaiming Tao, Philip L Tzou, Janin Nouhin, Ravindra K Gupta, Tulio de Oliveira, Sergei L Kosakovsky Pond, Daniela Fera, and Robert W Shafer. The biological and clinical significance of emerging sars-cov-2 variants. *Nature Reviews Genetics*, 22(12):757–773, 2021.

[34] Jiahui Chen and Guo-Wei Wei. Omicron ba. 2 (b. 1.1. 529.2): high potential for becoming the next dominant variant. *The journal of physical chemistry letters*, 13(17):3840–3849, 2022.

[35] Ivica Letunic and Peer Bork. Interactive tree of life (itol) v6: recent updates to the phylogenetic tree display and annotation tool. *Nucleic Acids Research*, page gkae268, 2024.

[36] Zhe Su, Xiang Liu, Layal Bou Hamdan, Vasileios Maroulas, Jie Wu, Gunnar Carlsson, and Guo-Wei Wei. Topological data analysis and topological deep learning beyond persistent homology-a review. *arXiv preprint arXiv:2507.19504*, 2025.
